# Circulating methylation level of *HTR2A* is associated with inflammation and disease activity in rheumatoid arthritis

**DOI:** 10.3389/fimmu.2022.1054451

**Published:** 2022-12-06

**Authors:** Jianan Zhao, Lingxia Xu, Cen Chang, Ping Jiang, Kai Wei, Yiming Shi, Linshuai Xu, Yixin Zheng, Yu Shan, Yanqin Bian, Li Li, Shicheng Guo, Steven J. Schrodi, Rongsheng Wang, Dongyi He

**Affiliations:** ^1^ Department of Rheumatology, Shanghai Guanghua Hospital, Shanghai University of Traditional Chinese Medicine, Shanghai, China; ^2^ Guanghua Clinical Medical College, Shanghai University of Traditional Chinese Medicine, Shanghai, China; ^3^ Institute of Arthritis Research in Integrative Medicine, Shanghai Academy of Traditional Chinese Medicine, Shanghai, China; ^4^ Arthritis Institute of Integrated Traditional and Western medicine, Shanghai Chinese Medicine Research Institute, Shanghai, China; ^5^ Computation and Informatics in Biology and Medicine, University of Wisconsin-Madison, Madison, WI, United States; ^6^ Department of Medical Genetics, School of Medicine and Public Health, University of Wisconsin-Madison, Madison, WI, United States

**Keywords:** rheumatoid arthritis, circulating methylation level, HTR2A, inflammation, disease activity

## Abstract

**Objectives:**

*HTR2A* is previously identified as a susceptibility gene for rheumatoid arthritis (RA). In this study, we performed the association analysis between DNA methylation of *HTR2A* with RA within peripheral blood samples.

**Methods:**

We enrolled peripheral blood samples from 235 patients with RA, 30 osteoarthritis (OA) patients, and 30 healthy controls. The DNA methylation levels of about *218 bp* from chr13: 46898190 to chr13: 46897973 (GRCh38/hg38) around *HTR2A* cg15692052 from patients were analyzed by targeted methylation sequencing.

**Results:**

We measured methylation status for 7 CpGs in the promoter region of *HTR2A* and obseved overall methylation status are signficantly increased in RA compared with normal inviduals (FDR= 9.05 x 10^-5^). The average cg15692052 methylation levels (methylation score) showed a positive correlation with CRP (r=0.15, *P*=0.023). Compared with the OA group or HC group, the proportion of haplotypes CCCCCCC (*FDR*=0.02 and 2.81 x 10^-6^) is signficantly increased while TTTTTCC (*FDR* =0.01) and TTTTTTT(*FDR* =6.92 x 10^-3^) are significantly decreased in RA. We find methylation haplotypes combining with RF and CCP could signficantly enhance the performance of the diagnosing RA and its comorbidities (hypertension, interstitial lung disease, and osteoporosis), especially in interstitial lung disease.

**Conclusions:**

In our study, we found signficant hypermethylation of promoter region of *HTR2A* which indicates the potential clinical diagnostic role in rheumatoid arthritis.

## Introduction

Rheumatoid arthritis(RA) is a chronic autoimmune disease that affects multiple organs and systems throughout the body (1). Risk factors for RA include the interaction of genetic, metabolic, and immune factors, and are complex pathological processes (2). Genetic factors are thought to be responsible for 50% of these patients, both genetic and epigenetic (1). Existing first-line drugs for RA mainly include disease-modifying antirheumatic drugs, nonsteroidal anti-inflammatory drugs, novel biologics, and hormonal drugs to maintain patients in disease remission or even low disease activity for an extended period. Although most patients have achieved some clinical efficacy with the application of multiple drug options, the presence of numerous disease heterogeneities has resulted in a poor response in some patients. Therefore, on the one hand, there is an urgent need to develop new therapeutic agents. On the other hand, early diagnosis and early intervention remain critical interventions for disease management, which can significantly improve the clinical prognosis and reduce the disability rate of patients (3, 4).

As previously mentioned, genetic factors are essential as pathological factors in RA, where genetic and environmental interactions lead to the emergence of epigenetics. Understanding the association and importance of epigenetics with RA is evolving with time, and its importance is indicated by the study of genome-wide epimodification maps generated by different cells and players in the context of the disease (5). Epigenetics mainly includes DNA methylation, micro RNA, and histone modifications (6). Among them, DNA methylation plays an important role in the occurrence and development of rheumatoid arthritis, which may be closely related to the pathogenesis of the disease and the efficacy of drugs (7–12). For example, defective DNA methylation of Treg cells leads to insufficient functional integrity (13). In contrast, methotrexate can restore impaired Treg cell function through demethylation of the *FOXP3* motif, leading to increased expression of *FOXP3* and *CTLA-4* and thus treating patients with RA (14).

The 5-hydroxytryptamine 2A (5-HT2A) serotonin receptor (*HTR2A*) is located on human chromosome 13q14-q21 and consists of three exons and five non-synonymous variants and two synonymous variants, and two introns containing more than 200 known variants (15). It primarily encodes the serotonin receptor 5-HT2A, a multi-acting neurotransmitter with current research focused on various emotional transmissions and psychiatric symptoms. For example, studies have found that rs6311 hypermethylation and rs6313 hypermethylation throughout the *HTR2A* promoter region in the brains of patients with schizophrenia and bipolar disorder may contribute to disease onset (16, 17). Increased rs6313 methylation in suicide attempters compared to peripheral leukocytes in bipolar and schizophrenic individuals without suicidal ideation (18). *HTR2A* expression is increased in patients with chronic fatigue syndrome and is regulated by methylation sites -1438, -1420, and -1224 in peripheral blood mononuclear cells (19). The study shows a high frequency of rs6313 TT genotype in the Japanese population and a low prevalence of RA (20, 21). There was a significant difference in the frequency of rs6313 (T102C polymorphism) between patients with RA and controls, and the frequency of TCT combinations was significantly lower in patients with RA. In comparison, the frequency of CTCT combinations was considerably higher (21). T cells from patients with RA with TC haplotype heterozygous for *HTR2A* produce higher levels of TNF-a, IL-6, and IFN-γ, and monocytes have higher levels of TNF-a in response to LPS stimulation. Still, cytokine production is inhibited by selective 5-HT2 receptor agonists (22). In addition, a gene-gene interaction between the protective haplotype in *HTR2A* and the *HLA-DRB1* shared epitope allele was suggested and correlated with RA autoantibody positivity (15). Thus, all of the above evidence suggests that genetic polymorphisms in the *HTR2A* are associated with susceptibility to RA and may affect the immune system in the context of RA through the 5-HT receptor system.

In this study, we collected peripheral blood samples from RA, osteoarthritis patients(OA), and healthy subjects(HC) for DNA methylation assay to clarify the correlation between DNA methylation alterations of *HTR2A* and RA. The aim is to provide an experimental basis and theoretical reference for the clinical discovery of diagnostic markers with practical value.

## Materials and methods

### Participants and peripheral blood collection

Guanghua Hospital Precision Medicine Research Cohort (PMRC) is a hospital-based longitudinal cohort to investigate risk factors, genetic susceptibility, pharmacogenetics for rheumatology diseases such as RA, osteoarthritis, and ankylosing spondylitis. From October 20 to November 30, 2021, we conducted participant recruitment at PMRC (235 patients with RA, 30 OA patients, and 30 healthy controls). The 2010 American college of rheumatology criteria serve as the inclusion standard for RA (15) (**Table S1**). All individuals’ clinical information was fully documented, and entire blood samples were taken. All research participants gave their informed permission and the guanghua hospital ethics committee authorized it (No. 2018-K-12).

### DNA methylation testing

The DNA methylation assay consists of sample quality control, polymerase chain reaction (PCR) primer design and optimization, heavy sulfite treatment, PCR reaction with specific labeling, and sequencing. Genomic DNA was first extracted from the peripheral blood of RA, OA, and HC, respectively. Sample quality control required concentrations ≥ 20 ng/μL, a total of ≥ 400 ng sample purity(OD260/280 = 1.7-1.9, OD260/230 ≥ 2.0). The primers were designed and optimized based on the “Methylation FastTarget V4.1” software. The primer sequences of primerF and primerR were GGGGTAGGAGGGTGGTAGG and TCACCACCTCTCTTCAAACAACTAC, respectively. The primers were amplified and subjected to heavy sulfite treatment. The unmethylated cytosine(C) of genomic DNA was converted to uracil (U). Then, the primers with Index sequence were used to introduce specific tag sequences compatible with the Illumina platform to the end of the library by PCR amplification. Finally, high-throughput sequencing was performed at Illumina Hiseq (Illumina, CA, USA) in 2×150 *bp* double-end sequencing mode to obtain FastQ data.

### Software packages and statistical analysis

Data visualization and statistical analysis using R packages including “tidyverse”, “ggplot2”, “ggstatsplot”. “Hmisc”, “PerformanceAnalytics”, “pROC”, “glmnet “, “rmda”, “RColorBrewer” (23). Using “tidyverse”, “ggplot2”, and “ggstatsplot” to determine the differential methylation levels and visualized (*FDR*<0.05). Correlations were calculated and visualized using “PerformanceAnalytics” and “Hmisc”. Using “pROC”, “glmnet”, “rmda”, “RColorBrewer “ to perform area under the receiver operating characteristic (AUROC) and construct logistic regressions to compare the effects of multiple factors with those of a single factor and visualize them.

## Results

### The methylation changes of the promoter region of HTR2A in RA compared with OA and normal

**Figure 1:**
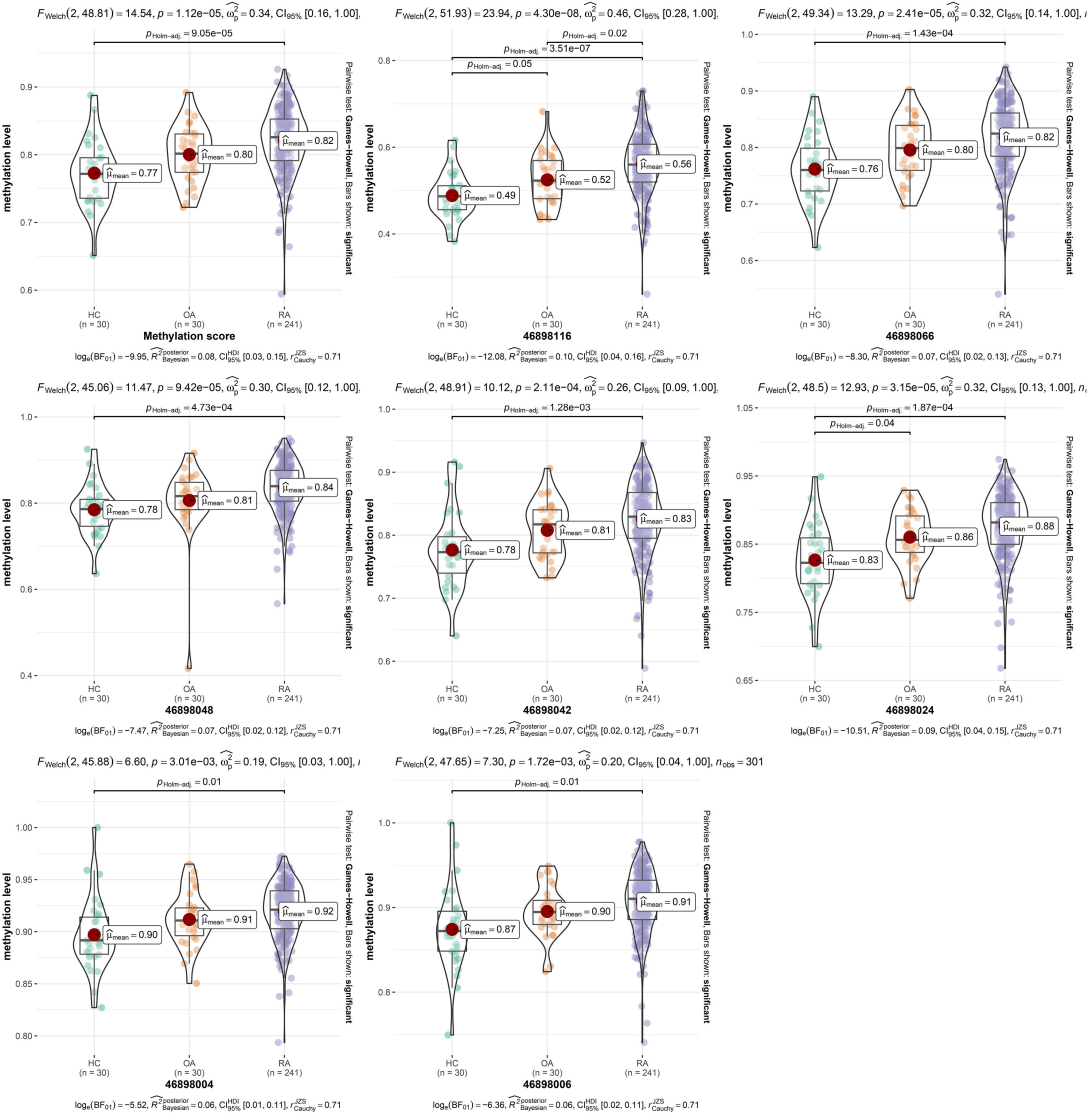
The methylation levels of different CpG sites. Comparison of methylation levels of different CpG sites of cg15692052 in RA, OA and HC groups using violin plot, FDR < 0.05 is statistically significant. RA, rheumatoid arthritis; OA, osteoarthritis patients; HC, healthy subjects.

In the *280bp* region near cg15692052, we detected a total of 7 CpGs sites, including 46898116, 46898066, 46898048, 46898042, 46898024, 46898006 and 46898004. Therefore, we plotted box plots to observe the proportional changes in methylation levels of CpGs sites within a *280bp* region near cg15692052 among the three groups. It showed that the average cg15692052 methylation levels were elevated in the RA group compared to the HC group (*FDR*=9.05 x 10^-5^) (**Figure 1**). Then we examined the methylation levels of different CpG sites. The results showed that the methylation levels of all seven CpG sites had an elevated trend compared with the HC group or the OA group. The methylation levels were significantly higher compared with the HC group (*FDR*=3.51 x 10^-7^, 1.43 x 10^-4^, 4.73 x 10^-4^, 1.28 x 10^-3^, 1.87 x 10^-4^,0.01 and 0.01). The methylation levels of 46898116 were also significantly higher compared with the OA group (*FDR*=0.02) (**Figure 1**).

### Correlation analysis between HTR2A methylation with common clinical characteristics

**Figure 2:**
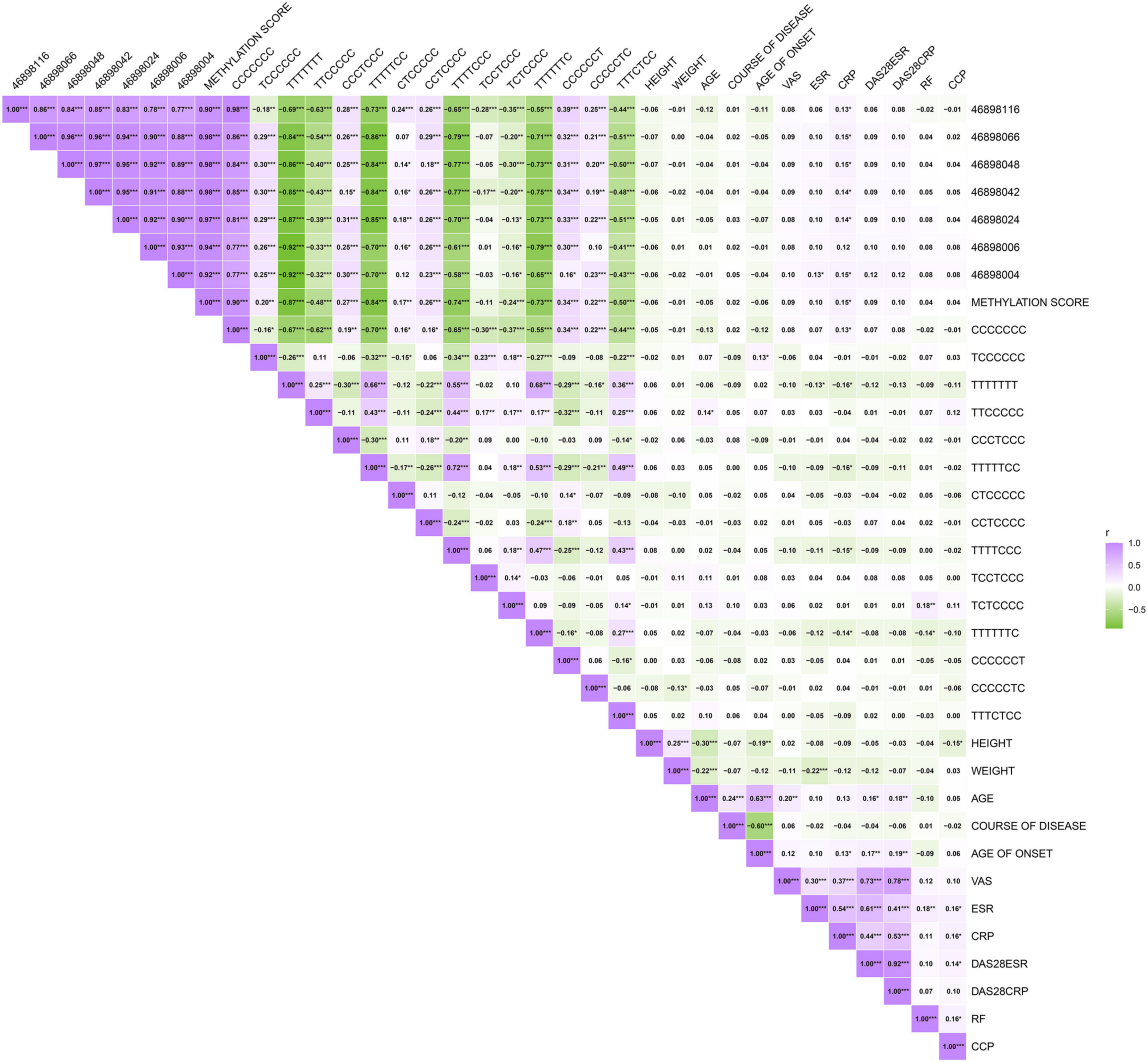
The correlation of CpGs sites and different haplotypes with interclinical indicator. Correlation plots were used to observe the correlations. ***P < 0.001, **P < 0.01, *P < 0.05.

We first examined the pearson correlation between different CpGs sites within this region. We found a correlation between various CpGs sites, including the methylation score calculated from the mean of all CpGs (**Figure 2**). We further examined the correlation between methylation levels of different CpGs sites and common clinical indicators, including CCP, CRP, ESR, RF, the disease activity score-28 with ESR (DAS28-ESR), and the disease activity score-28 with CRP (DAS28-CRP). 46898116, 46898066, 46898048, 46898042, 46898024, 46898004, and methylation score showed a significant positive correlation with CRP(*P*=0.045, 0.017, 0.020, 0.033, 0.025, 0.024, and 0.023).46898004 showed a significant positive correlation with ESR (*P*=0.044) (**Figure 2**).

### HTR2A methylation combines with RF and CCP enhance the diagnostic accuracy

**Figure 3:**
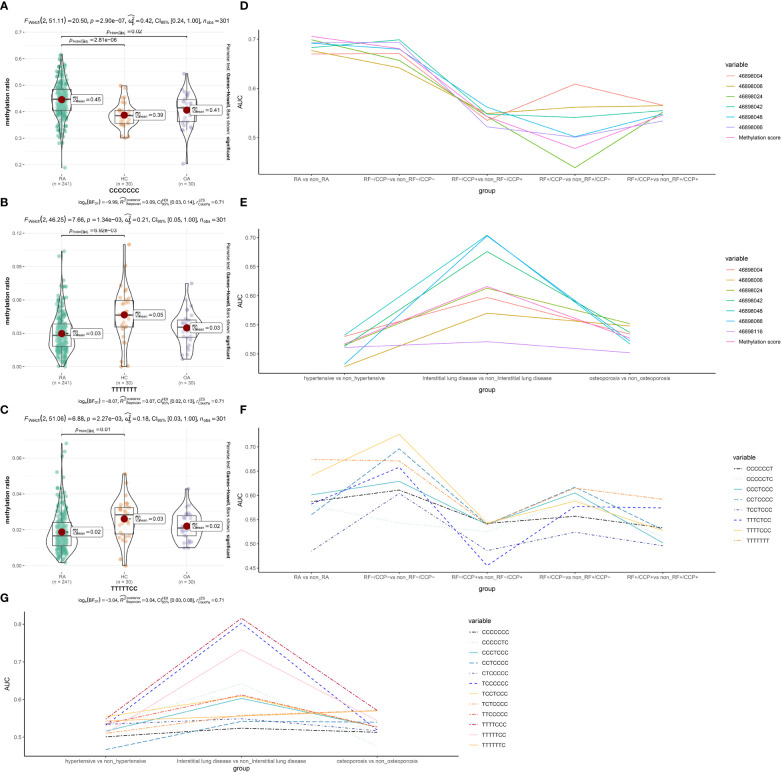
Specificity and accuracy of different CpG sites and haplotypes combined with RF or CCP for the diagnosis of RA and RA-related complications. **(A-C)** Haplotypes with significant differences **(D-G)** different CpG sites and haplotypes combined with RF or CCP for the diagnosis of RA-related complications. *FDR <*0.05 is statistically significant. RA, rheumatoid arthritis; OA, osteoarthritis patients; HC, healthy subjects.

We then investigated the specificity and accuracy of different CpG sites diagnosed alone and in combination with CCP or RF. First, we set five subgroups, whether RA, RF-/CCP-, RF-/CCP+, RF+/CCP- and RF+/CCP+, then we constructed logistic models and calculated the AUC, which often represents the specificity and accuracy of the diagnosis (**Figures 3D–G** and **Table S2**). We plotted the line graphs and found that these individual CpGs sites had the highest accuracy for the diagnosis of RA as well as RF-/CCP- (**Figures 3D–E**).

**Figure 4:**
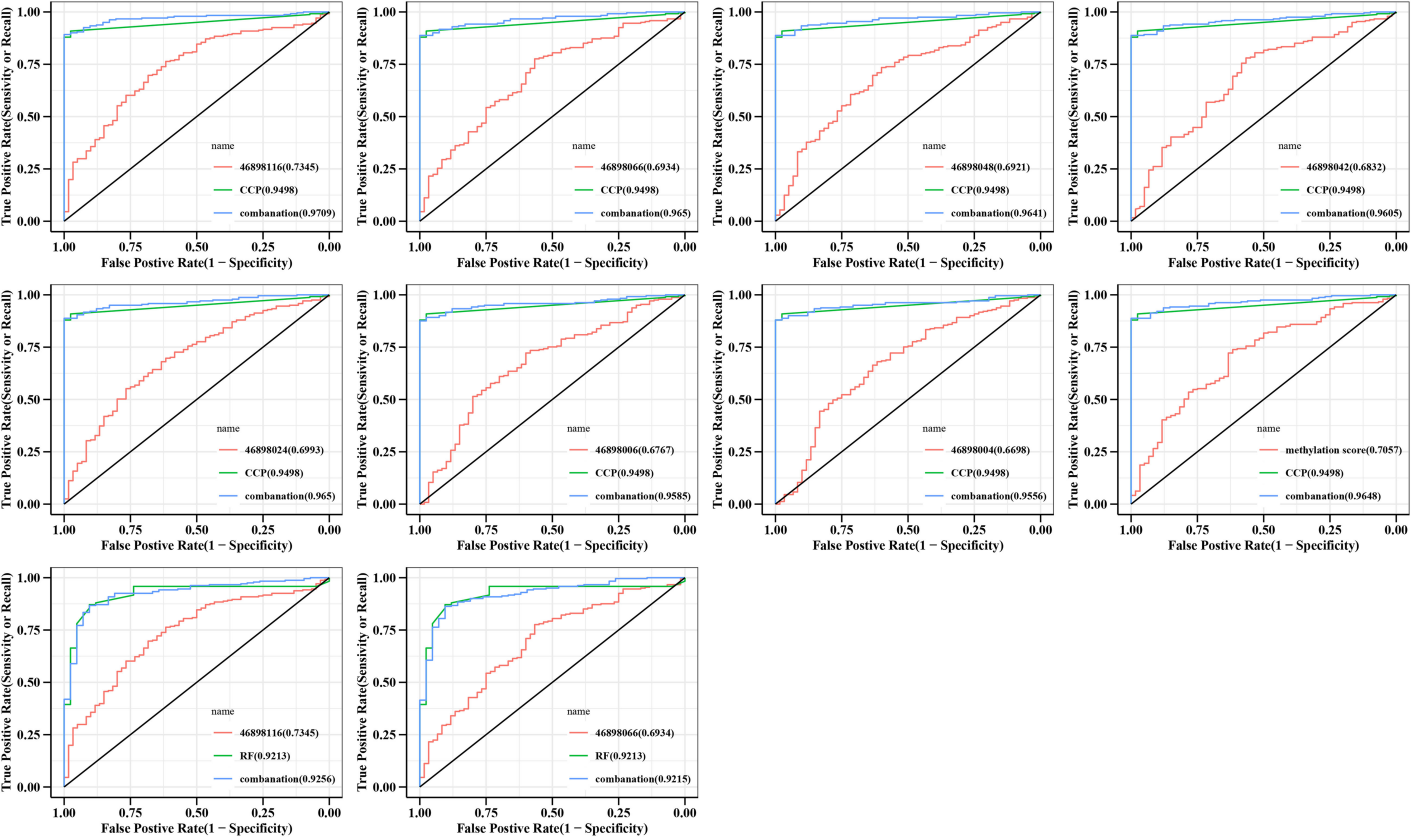
Specificity and accuracy of different CpG sites combined with RF or CCP for the diagnosis of RA. Logstic regression models were constructed and ROC curves were plotted, with the AUC area representing the accuracy and specificity of the diagnosis. CCP, anti-cyclic peptide containing citrulline; RF, rheumatoid factor; combanation, different CpG sites combined with RF or CCP.

RF and CCP are important indicators for a diagnosis of RA. Our data showed that the AUC curve areas of RF and CCP alone were 0.921 (95% CI 0.882-0.961) and CCP 0.950 (95% CI 0.928-0.971), respectively. The diagnostic sensitivity of CCP was enhanced when combined with different CpGs sites with AUC curve areas of 0.971 (95% CI 0.953-0.989), 0.965 (95% CI 0.946-0.984), 0.964 (95% CI 0.944-0.984), 0.961 (95% CI 0.940-0.981), 0.965 (95% CI 0.946-0.984), 0.959 (95% CI 0.937-0.980), 0.956 (95% CI 0.933-0.978), respectively (**Figure 4**). For RF. the sensitivity was enhanced only when combined with 46898116 and 46898066 with AUC curve areas of 0.926 (95% CI 0.887-0.964),0.921 (95% CI 0.882-0.960), respectively (**Figure 4**).

### Haplotype analysis to HTR2A among RA, OA and normal blood samples

We found that there are 15 haplotypes in a 280bp region near cg15692052. Compared with the OA group or HC group, the proportion of haplotypes CCCCCCC increased significantly (*FDR*=0.02 and 2.81 x 10^-6^). Compared with the HC group, TTTTTCC (*FDR* =0.01) and TTTTTTT(*FDR* =6.92 x 10^-3^) decreased significantly. The other haplotypes were not significantly different. (**Figures 3A–C)**

There are multiple haplotypes within a *280bp* region near cg15692052 in which there are abnormal changes in the proportion. There is also a correlation between them (**Figure 2**). There were positive correlations between CCCCCCC and CRP (*P*=0.050). TCCCCCC had a positive correlation with age of onset (*P*=0.043). TTCCCCC had a positive correlation with age (*P*=0.037). TTTTTTT showed a negative correlation with CRP and ESR (*P*=0.011 and 0.040). TTTTTCC and TTTTCCC had a negatively correlate with CRP (*P*=0.012 and 0.021). TCTCCCC has a positive correlation with RF (*P*=0.005). TTTTTTC was negatively correlated with both RF and CRP (*P*=0.030, and 0.033). CCCCCTC has a negative correlation with body weight (*P*=0.043) (**Figure 2**).

### Methylation haplotype shows diagnostic performance of RF and CCP

We then investigated the specificity and accuracy of different haplotypes diagnosed alone (**Table S3**) and in combination with CCP or RF (**Figure S1**). The grouping was the same as in 3.3, with similar trends to the different CpGs sites, and most haplotypes were diagnostically more accurate for RA and RF-/CCP-. We then still combined the different haplotypes combined with RF and CCP separately. The diagnostic sensitivity of CCP could be enhanced, including CCCCCCC (0.969, 95% CI 0.951 to 0.988), TCCCCCC (0.957, 95% CI 0.935~0.979), TTTTTTT (0.956, 95% CI 0.934~0.978), CCCTCCC (0.956, 95% CI 0.933~0.978), TTTTTCC (0.962, 95% CI 0.942~0.983), CTCCCCC (0.963, 95% CI 0.943~0.983), CCTCCCC (0.955, 95% CI 0.932~0.978), TTTTCCC (0.956, 95% CI 0.934~0.978), TCTCCCC (0.951, 95% CI 0.926~0.976), TTTTTTC (0.958, 95% CI 0.937 to 0.980), CCCCCCT(0.956, 95% CI 0.934 to 0.978), CCCCCTC (0.959, 95% CI 0.937 to 0.980), TTTCTCC (0.967, 95% CI 0.949 to 0.986) (**Figure S1**). Only RF combined with CCCCCCC enhanced the diagnostic sensitivity of RF with an AUC of 0.924 (95% CI 0.886-0.963) (**Figure S1**).

### Methylation loci and haplotype could enhance the diagnosis of RA-related complications

RA is often associated with multiple comorbidities. We further analyzed the methylation levels of different CpG sites and the proportion of different haplotypes for the diagnostic accuracy of three common RA comorbidities, including hypertension, interstitial lung disease, and osteoporosis (**Table S4**-**5**). Both were more accurate for diagnosing interstitial lung disease (**Figures 3E, G** **).**

## Discussion

*HTR2A* primarily encodes the G protein-coupled serotonin 5-HT2A receptor, and the 5-HT/serotonin receptor system has been shown to be associated with inflammatory responses in addition to psychiatric disorders. The role of psychological factors in RA has been discussed in several articles (24–26). In short, psychological factors may influence the outcome and degree of patients’ pathology. Patients with RA in clinical settings also often suffer from several psychological and psychiatric disorders, such as depression, due to long-term chronic pain. As shown previously, genetic variants and methylation alterations in *HTR2A* have been shown to have a significant role in various psychiatric disorders. Therefore, in the context of RA, in conjunction with abnormal mental health changes in RA patients, it is also an important research direction to investigate its association with the psychological factors of RA in depth in the future. In addition, as mentioned previously, *HTR2A* has also been shown to be involved in abnormal inflammatory responses. For example, the 5-HT2A receptor and signal transducer and activator of transcription 3 (STAT3)/Janus kinase 2 (JAK2) are interconnected and jointly activate intracellular phosphorylation pathways (27). 5-HT can also affect cytokine production in LPS-stimulated peripheral blood mononuclear cells, including the inhibition of TNF-a (28, 29). In the present study, we measured the altered DNA methylation of *HTR2A* in RA peripheral blood. First, we found that the average methylation level of cg15692052 of *HTR2A* and the methylation levels of different CpG sites were elevated, and in general, DNA methylation often implies silencing or reduction of gene expression. Our previous study found a significantly low expression of *HTR2A* in RA fibroblast-like synoviocytes (*q.value*=0.012, logFC=-0.73) (30). This is also consistent with the experimental results of our current assay. Our sequenced *280bp* range is between 5-UTR and promoter, therefore, the methylation changes may also affect gene expression. The reduced expression of *HTR2A* may lead to an imbalance of anti-inflammatory mechanisms promoting inflammation in RA.

We also correlated with common RA clinical indicators to investigate the effect of methylation levels of different CpG sites and inflammation. ESR and CRP are widely used as laboratory indicators of common clinical inflammatory activity in RA. We found that the methylation levels of all seven CpG sites showed a significant positive correlation with ESR or CRP, which further validates that high methylation levels may lead to low expression of genes and thus promote inflammation. We also found that the methylation levels of CpG sites (46898116,46898066,46898048,46898042,46898024, 46898004, and methylation score) showed a positive correlation with CCP, respectively, supporting these results. In addition, the presence of autoantibodies, including RF and CCP, are characteristic markers of RA and may assist in diagnosing RA. Our data also support this result, with AUCs of 0.921 and 0.950 for RF and CCP alone to diagnose RA, respectively. Further, we analyzed the accuracy of methylation levels of different CpG sites alone in diagnosing RA. Our results show that although the diagnostic accuracy of CpGs or methylated haplotypes combined with RF or CCP is less improved, improving the clinical diagnosis of patients with RF and CCP serologically negative RA is more helpful. Because patients with RF and CCP negative RA lack a valid clinical diagnosis, and approximately 1/3 of patients have normal RF and CCP clinically (serologically negative) (31).

We further identified that cg15692052 methylation has multiple haplotypes and the proportions vary significantly in RA, OA, and HC. The changes in the proportions of different methylation haplotypes may indicate the disease’s level of inflammation. The haplotype responds to the combined alteration of seven CpGs, which may reflect a methylation pattern. We first found that almost all individual CpGs are hypermethylated at high levels. In contrast, the proportion of the combined form TTTTTTT, which represents all of the non-methylation, is also significantly lower in RA. The proportion of the form CCCCCC, which means all of the methylations, is also significantly elevated, basically consistent with the analysis of individual CpGs. Second, when some or all of the seven CpGs are methylated, there may be a potential impact on the expression of the gene itself and thus affect RA. But the exact direction of the change in gene expression may be unknown and needs to be analyzed in conjunction with our future studies. Also, we further jointly analyzed the correlation of these proportional changes with common clinical indicators in RA. It found that the proportions of TTTTTCC, TTTTCCC, TTTTTTT, and TTTTTTC haplotypes showed a negative correlation with common inflammatory indicators. It suggests that these haplotypes may be associated with lower levels of inflammation. CCCCCCC was increased in RA and showed positive correlations with CRP, suggesting that such haplotypes may mean higher levels of inflammation. Our further analysis of the different haplotypes of cg15692052 methylation alone can help the disease diagnosis of RA. Still, combined with CCP or RF, higher diagnostic accuracy can be obtained, but only CCCCCCC can enhance the accurate specificity of RF. Therefore, a flexible combination is needed for the diagnosis of RA.

RA also has a variety of clinical comorbidities, such as an increased risk of subclinical vascular disease (32). Michael et al. elucidated the impact of concomitant cardiovascular disease in RA, with the highest prevalence of hypertension at 73% in RA patients (33). Multiple pro-inflammatory factors of RA also increase the incidence of combined hypertension in patients (34). However, patients with RA complicated by hypertension are not adequately diagnosed and treated. For example, in a study of 400 patients with RA, Panoulas et al. found that 282 patients with RA had concomitant hypertension, but only 171 received antihypertensive therapy, of which only 37 were effectively controlled. 111 were not diagnosed and treated (35). Bone loss in patients with RA can occur locally, affecting inflamed joints and throughout the body, leading to osteoporosis and significantly increasing the burden of fracture and disability in patients with RA (36). This may often be associated with glucocorticoid use, systemic inflammation, and impaired bone mass (31). The diagnosis and treatment of RA combined with osteoporosis should be based on early diagnosis, careful assessment of adverse events with current therapies, and effective intervention (37). Therefore, diagnosing RA combined with hypertension or osteoporosis is critical. Although our data show that different CpGs and methylation haplotypes are not highly accurate for both, they also have implications for clinical diagnosis and intervention. Interstitial lung disease is also a serious complication of RA, with a significantly increased mortality rate compared to patients with RA without interstitial lung disease (38). Current diagnostic methods for RA combined with interstitial lung disease are limited and mainly X-ray electron computed tomography. We found that different haplotypes of cg15692052 methylation can assist in diagnosing RA comorbidity with exceptionally high specificity for interstitial lung disease. This is critical for early detection and intervention in RA combined with interstitial lung disease to reduce mortality.

In conclusion, we found elevated methylation levels of *HTR2A* cg15692052 and different CpGs sites in RA. It may be associated with reduced gene expression of *HTR2A* and correlated with common clinical indicators such as RF, CCP, ESR, and CRP. It may assist in diagnosing clinical RA, suggesting that abnormal changes in methylation levels of *HTR2A* are associated with the immune inflammation of RA. In addition, the presence of differences in the ratio of different methylation haplotypes in *HTR2A* cg15692052 is also correlated with common clinical indicators such as RF. It can assist in the clinical diagnosis of RA. It is worth noting that different CpGs sites and haplotypes of *HTR2A* cg15692052 can assist in diagnosing RA-related complications, especially for interstitial lung disease. However, there are undeniably some limitations of our study: first, our study was based on the analysis of DNA extracted from peripheral blood mononuclear cells of RA patients, where the presence of cellular heterogeneity may be a confounding factor in the experiment, and our results show that the overall DNA methylation levels are hypermethylated, but the specifics of which cells are specific still need further elucidation to clarify the different intrinsic differences in DNA methylation in cellular contexts, such as T lymphocytes, B lymphocytes (8–11). Second, for the correlation analysis in the results, although there is a significant difference, but presents a weak correlation; undeniably, this may be a trend of weak linear correlation. In the future, we will further optimize the experimental conditions and increase the cell-specific background in-depth study.In summary, this study showed multiple associations of abnormal methylation changes of *HTR2A* cg15692052 with RA and had the potential as a future clinical diagnostic marker for RA.

## Data availability statement

The datasets presented in this study can be found in online repositories. The names of the repository/repositories and accession number(s) can be found below: https://github.com/aaron-jianan/methylation.git, Github.

## Ethics statement

The studies involving human participants were reviewed and approved by the guanghua hospital ethics committee authorized it (No. 2018-K-12). The patients/participants provided their written informed consent to participate in this study.

## Author contributions

JZ is responsible for the collection, collation, and writing of the original manuscript. LXX, CC, PJ, KW, YiS, LSX, YZ, YuS, YB, LL, SG and SS for the collection, collation of the original data. RW and DH are responsible for the concept development, revision, and manuscript review. All authors contributed to the article and approved the submitted version.

## Supplementary material

Supplementary Figure 1 Specificity and accuracy of the proportion of haplotypes of cg15692052 in combination with RF or CCP for the diagnosis of RA. Logstic regression models were constructed and ROC curves were plotted, with the AUC area representing the accuracy and specificity of the diagnosis. CCP, anti-cyclic peptide containing citrulline; RF, rheumatoid factor;combanation, different haplotypes combined with RF or CCP.
