# Supplementary figures and images for: Circulating methylation level of HTR2A is associated with inflammation and disease activity in rheumatoid arthritis
Source: Front Immunol. 2022 Dec 6;13:1054451. doi: 10.3389/fimmu.2022.1054451 (PMC9763304; doi:10.3389/fimmu.2022.1054451)

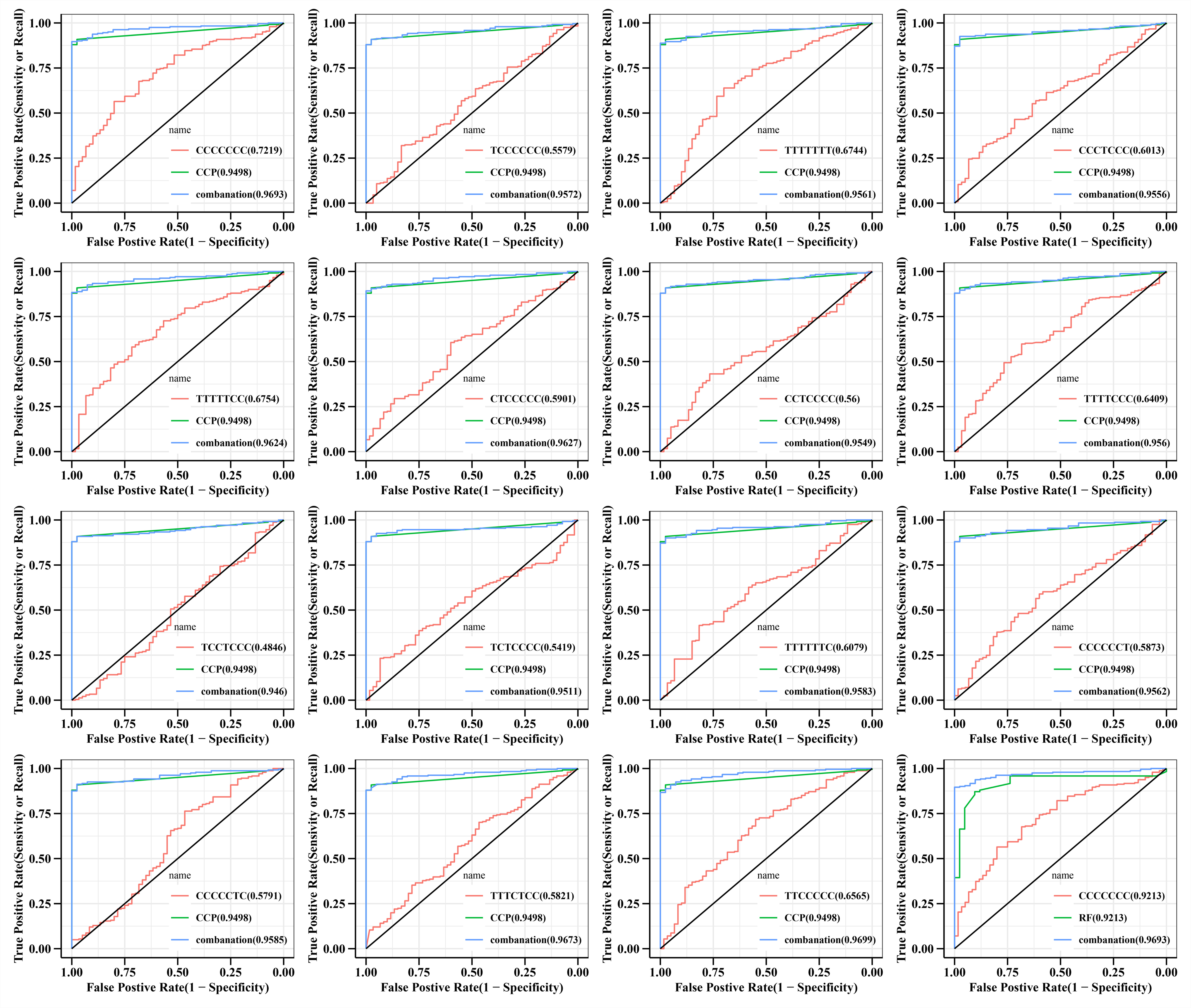

Supplement: Supplementary Figure 1 — Specificity and accuracy of the proportion of haplotypes of cg15692052 in combination with RF or CCP for the diagnosis of RA. Logstic regression models were constructed and ROC curves were plotted, with the AUC area representing the accuracy and specificity of the diagnosis. CCP, anti-cyclic peptide containing citrulline; RF, rheumatoid factor;combanation, different haplotypes combined with RF or CCP. [file Image_1.tif]
