# Supplementary material for: Circulating methylation level of HTR2A is associated with inflammation and disease activity in rheumatoid arthritis
Source: Front Immunol. 2022 Dec 6;13:1054451. doi: 10.3389/fimmu.2022.1054451 (PMC9763304; doi:10.3389/fimmu.2022.1054451)
Supplement: Supplementary file 3 [file DataSheet_1.pdf]

[DAS28-CRP]

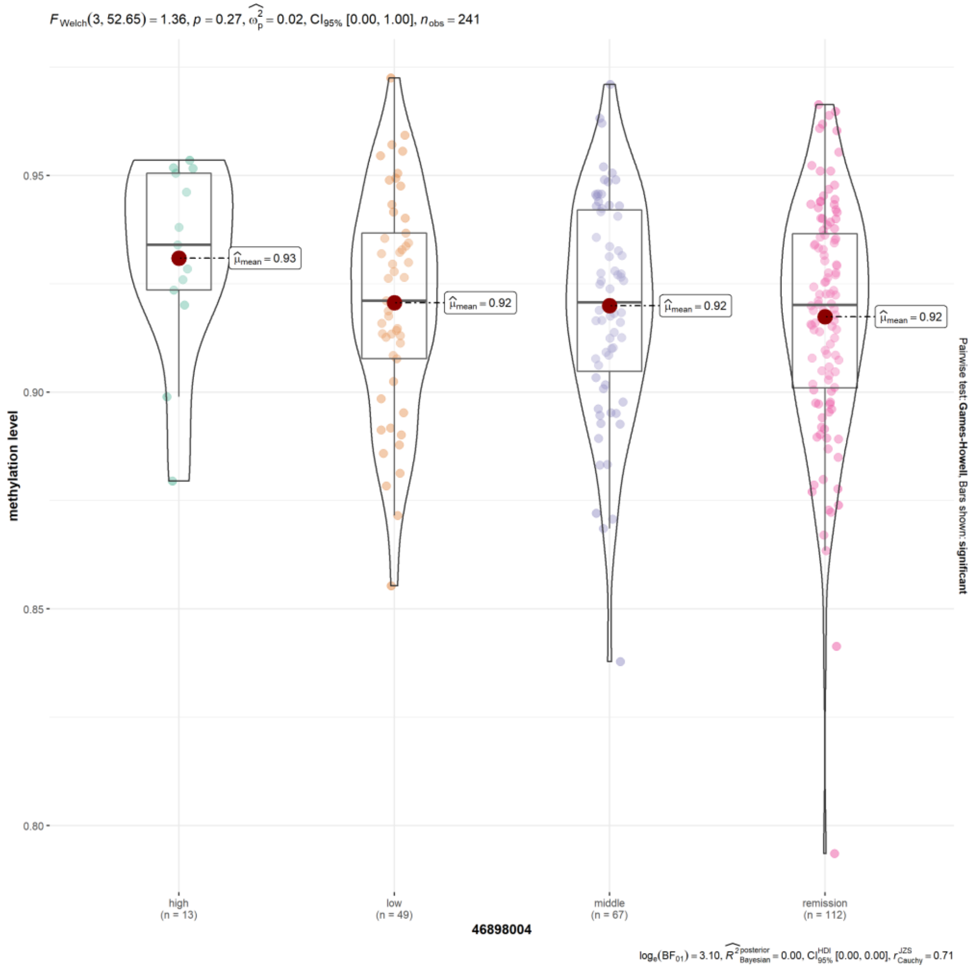
$F_{Welch}(3, 52.65) = 1.36, p = 0.27, \widehat{\omega_p^2} = 0.02, CI_{95\%}$ [0.00, 1.00], $n_{obs} = 241$
methylation level
0.95
0.90
0.85
0.80
$\hat{\mu}_{mean} = 0.93$
$\hat{\mu}_{mean} = 0.92$
$\hat{\mu}_{mean} = 0.92$
$\hat{\mu}_{mean} = 0.92$
high
(n = 13)
low
(n = 49)
middle
(n = 67)
remission
(n = 112)
46898004
Pairwise test: Games-Howell; Bars shown: significant
$\log_e(BF_{01}) = 3.10, \widehat{R^2_{Bayesian}}^{posterior} = 0.00, CI_{95\%}^{HDI}$ [0.00, 0.00], $r_{Cauchy}^{JZS} = 0.71$

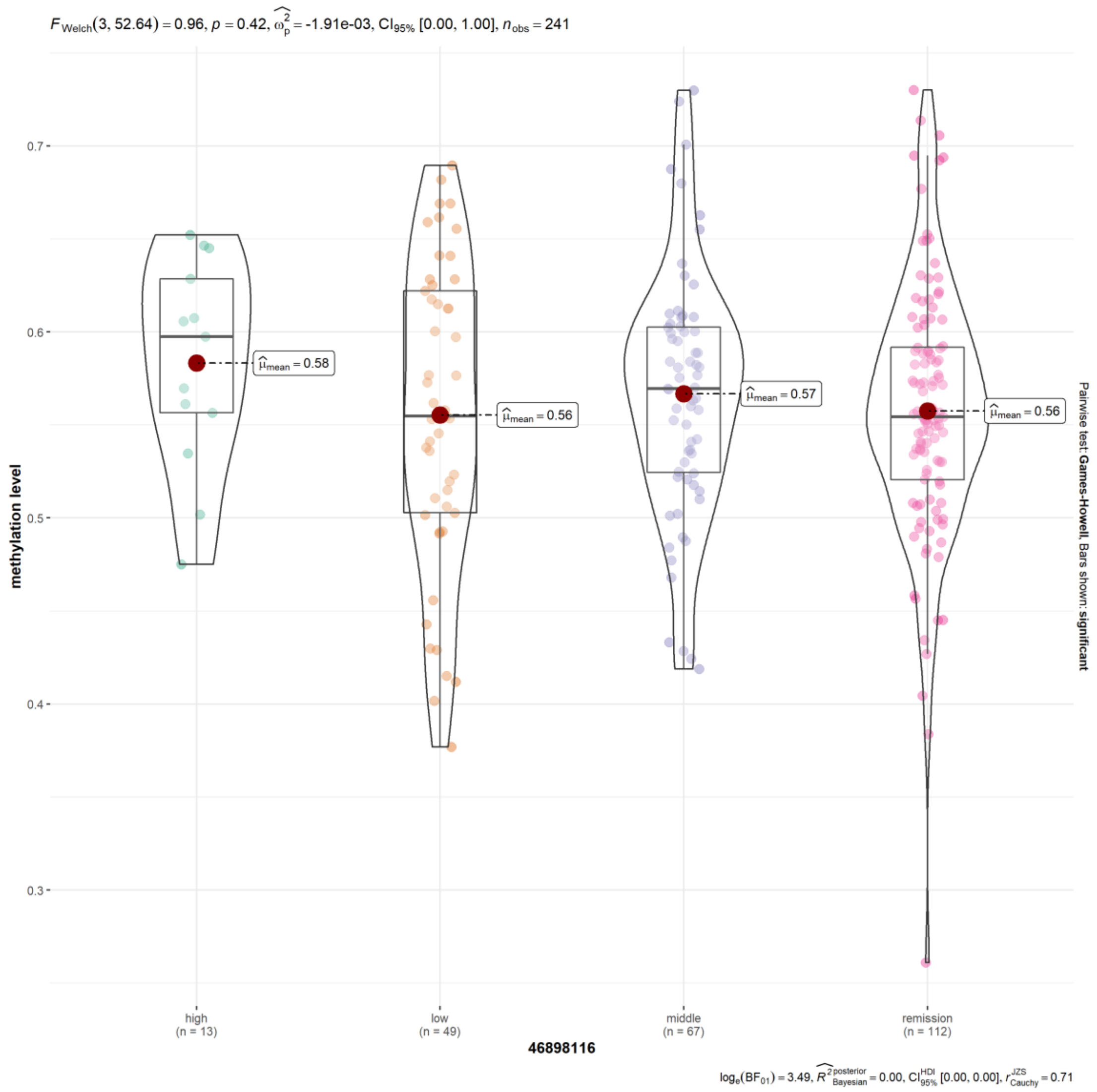
$F_{Welch}(3, 52.64) = 0.96, p = 0.42, \widehat{\omega_p^2} = -1.91e-03, CI_{95\%} [0.00, 1.00], n_{obs} = 241$
methylation level
0.7
0.6
0.5
0.4
0.3
$\hat{\mu}_{mean} = 0.58$
$\hat{\mu}_{mean} = 0.56$
$\hat{\mu}_{mean} = 0.57$
$\hat{\mu}_{mean} = 0.56$
high (n = 13)
low (n = 49)
middle (n = 67)
remission (n = 112)
46898116
Pairwise test: Games-Howell, Bars shown: significant
$\log_e(BF_{01}) = 3.49, \widehat{R^2}^{posterior}_{Bayesian} = 0.00, CI^{HDI}_{95\%} [0.00, 0.00], r^{JZS}_{Cauchy} = 0.71$

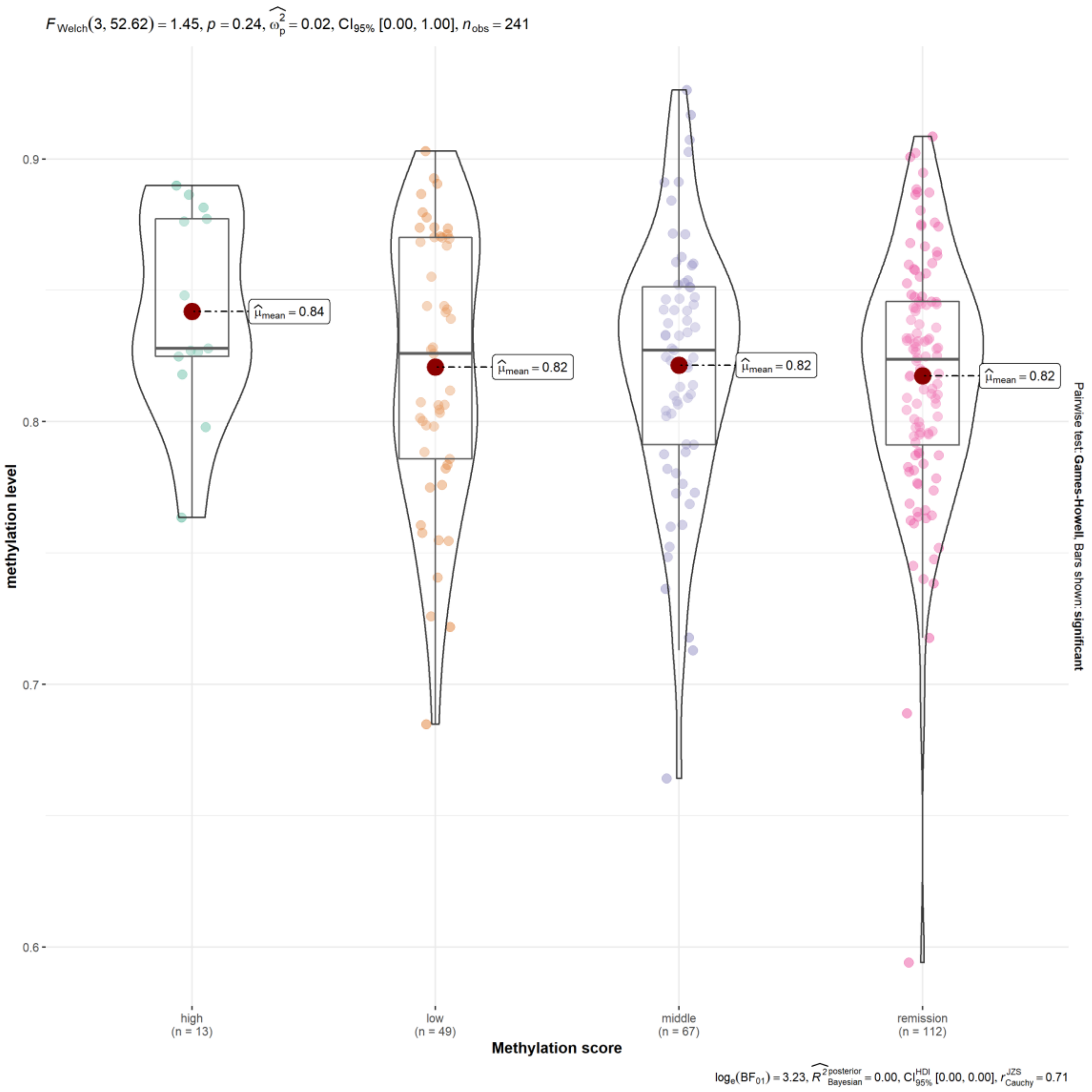

$F_{Welch}(3, 52.62) = 1.45$, $p = 0.24$, $\widehat{\omega_p^2} = 0.02$, $CI_{95\%}$ [0.00, 1.00], $n_{obs} = 241$
$\hat{\mu}_{mean} = 0.84$
$\hat{\mu}_{mean} = 0.82$
$\hat{\mu}_{mean} = 0.82$
$\hat{\mu}_{mean} = 0.82$
0.9
0.8
0.7
0.6
methylation level
high
(n = 13)
low
(n = 49)
middle
(n = 67)
remission
(n = 112)
Methylation score
Pairwise test: Games-Howell, Bars shown: significant
$\log_e(BF_{01}) = 3.23$, $\widehat{R^2}^{posterior}_{Bayesian} = 0.00$, $CI^{HDI}_{95\%}$ [0.00, 0.00], $r^{JZS}_{Cauchy} = 0.71$

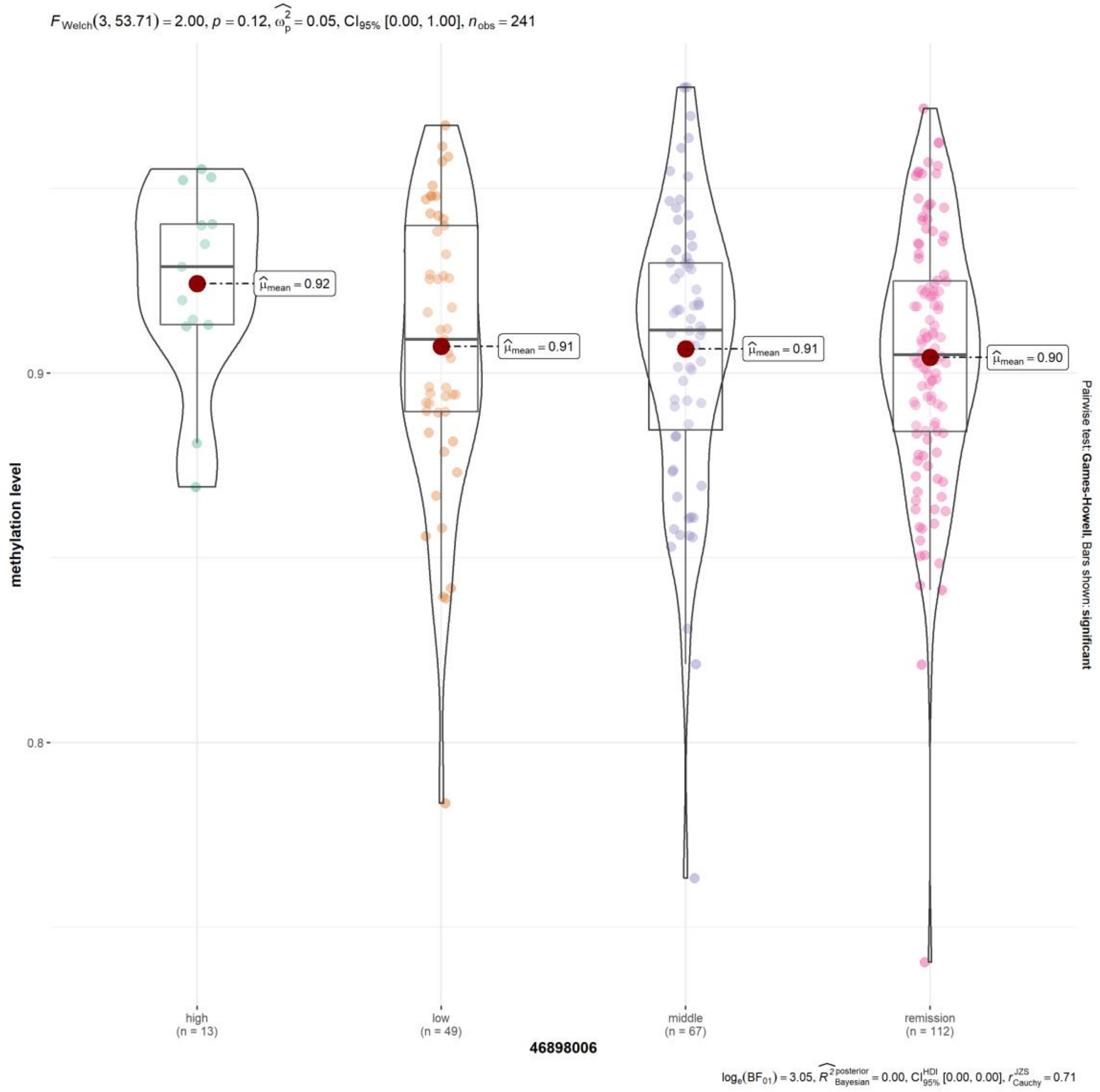

$F_{\text{Welch}}(3, 53.71) = 2.00$, $p = 0.12$, $\widehat{\omega_p^2} = 0.05$, $\text{CI}_{95\%}$ [0.00, 1.00], $n_{\text{obs}} = 241$
methylation level
0.9
0.8
$\widehat{\mu}_{\text{mean}} = 0.92$
$\widehat{\mu}_{\text{mean}} = 0.91$
$\widehat{\mu}_{\text{mean}} = 0.91$
$\widehat{\mu}_{\text{mean}} = 0.90$
high (n = 13)
low (n = 49)
middle (n = 67)
remission (n = 112)
46898006
Pairwise test: Games-Howell; Bars shown: significant
$\log_e(\text{BF}_{01}) = 3.05$, $\widehat{R^2}^{\text{posterior}}_{\text{Bayesian}} = 0.00$, $\text{CI}^{\text{HDI}}_{95\%}$ [0.00, 0.00], $r^{\text{JZS}}_{\text{Cauchy}} = 0.71$

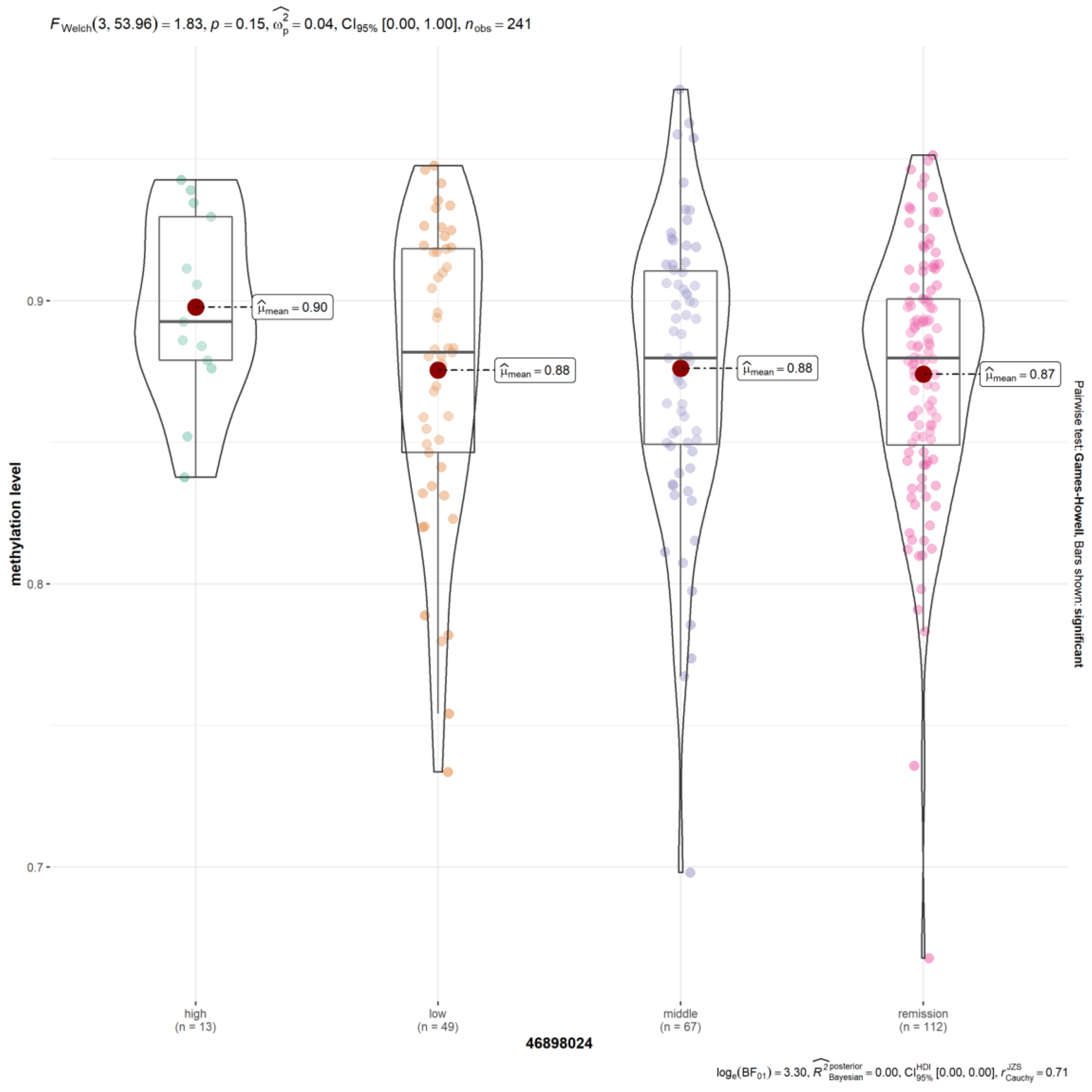

$F_{Welch}(3, 53.96) = 1.83$, $p = 0.15$, $\widehat{\omega_p^2} = 0.04$, $CI_{95\%}$ [0.00, 1.00], $n_{obs} = 241$
$\widehat{\mu}_{mean} = 0.90$
$\widehat{\mu}_{mean} = 0.88$
$\widehat{\mu}_{mean} = 0.88$
$\widehat{\mu}_{mean} = 0.87$
0.9
0.8
0.7
methylation level
high (n = 13)
low (n = 49)
middle (n = 67)
remission (n = 112)
46898024
Pairwise test: Games-Howell; Bars shown: significant
$\log_e(BF_{01}) = 3.30$, $\widehat{R^2}^{posterior}_{Bayesian} = 0.00$, $CI^{HDI}_{95\%}$ [0.00, 0.00], $r^{JZS}_{Cauchy} = 0.71$

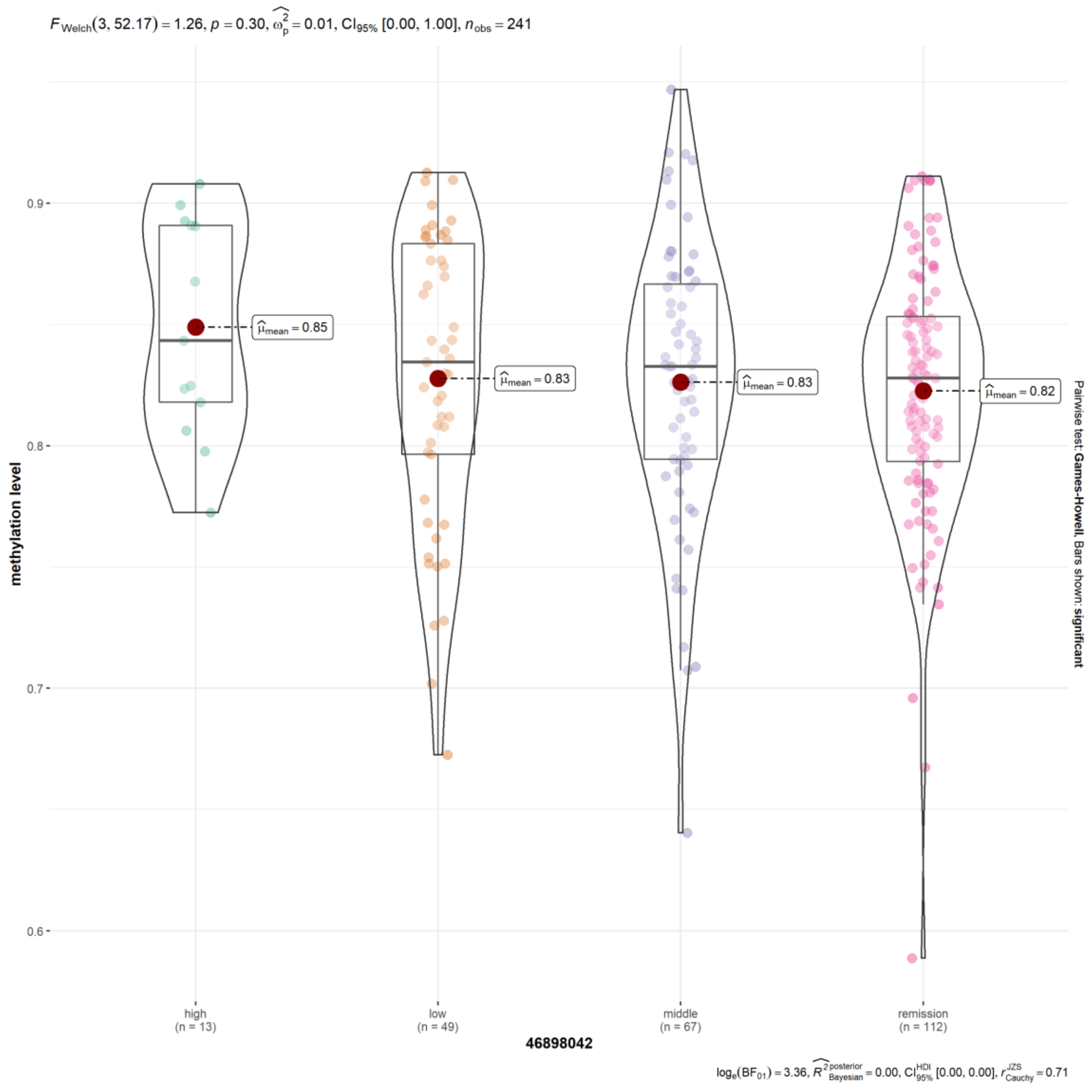

$F_{Welch}(3, 52.17) = 1.26$, $p = 0.30$, $\widehat{\omega_p^2} = 0.01$, $CI_{95\%}$ [0.00, 1.00], $n_{obs} = 241$
methylation level
0.9
0.8
0.7
0.6
$\hat{\mu}_{mean} = 0.85$
$\hat{\mu}_{mean} = 0.83$
$\hat{\mu}_{mean} = 0.83$
$\hat{\mu}_{mean} = 0.82$
high (n = 13)
low (n = 49)
middle (n = 67)
remission (n = 112)
46898042
Pairwise test: **Games-Howell**; Bars shown: **significant**
$\log_e(BF_{01}) = 3.36$, $\widehat{R^2}^{posterior}_{Bayesian} = 0.00$, $CI_{95\%}^{HDI}$ [0.00, 0.00], $r_{Cauchy}^{JZS} = 0.71$

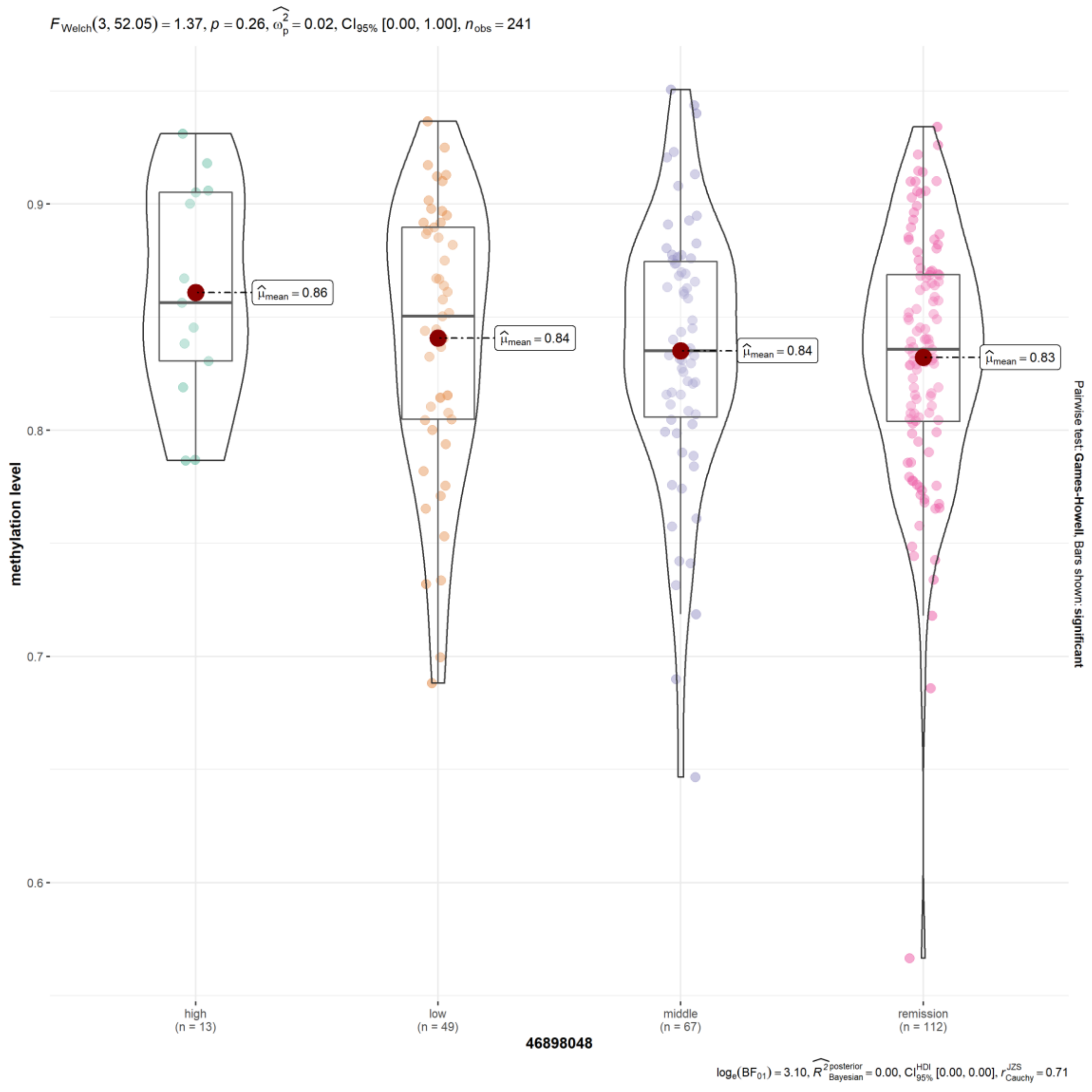

$F_{Welch}(3, 52.05) = 1.37$, $p = 0.26$, $\widehat{\omega_p^2} = 0.02$, $CI_{95\%}$ [0.00, 1.00], $n_{obs} = 241$
methylation level
0.9
0.8
0.7
0.6
$\hat{\mu}_{mean} = 0.86$
$\hat{\mu}_{mean} = 0.84$
$\hat{\mu}_{mean} = 0.84$
$\hat{\mu}_{mean} = 0.83$
high
(n = 13)
low
(n = 49)
middle
(n = 67)
remission
(n = 112)
46898048
Pairwise test: **Games-Howell**; Bars shown: **significant**
$\log_e(BF_{01}) = 3.10$, $\widehat{R^{2}}^{posterior}_{Bayesian} = 0.00$, $CI^{HDI}_{95\%}$ [0.00, 0.00], $r^{JZS}_{Cauchy} = 0.71$

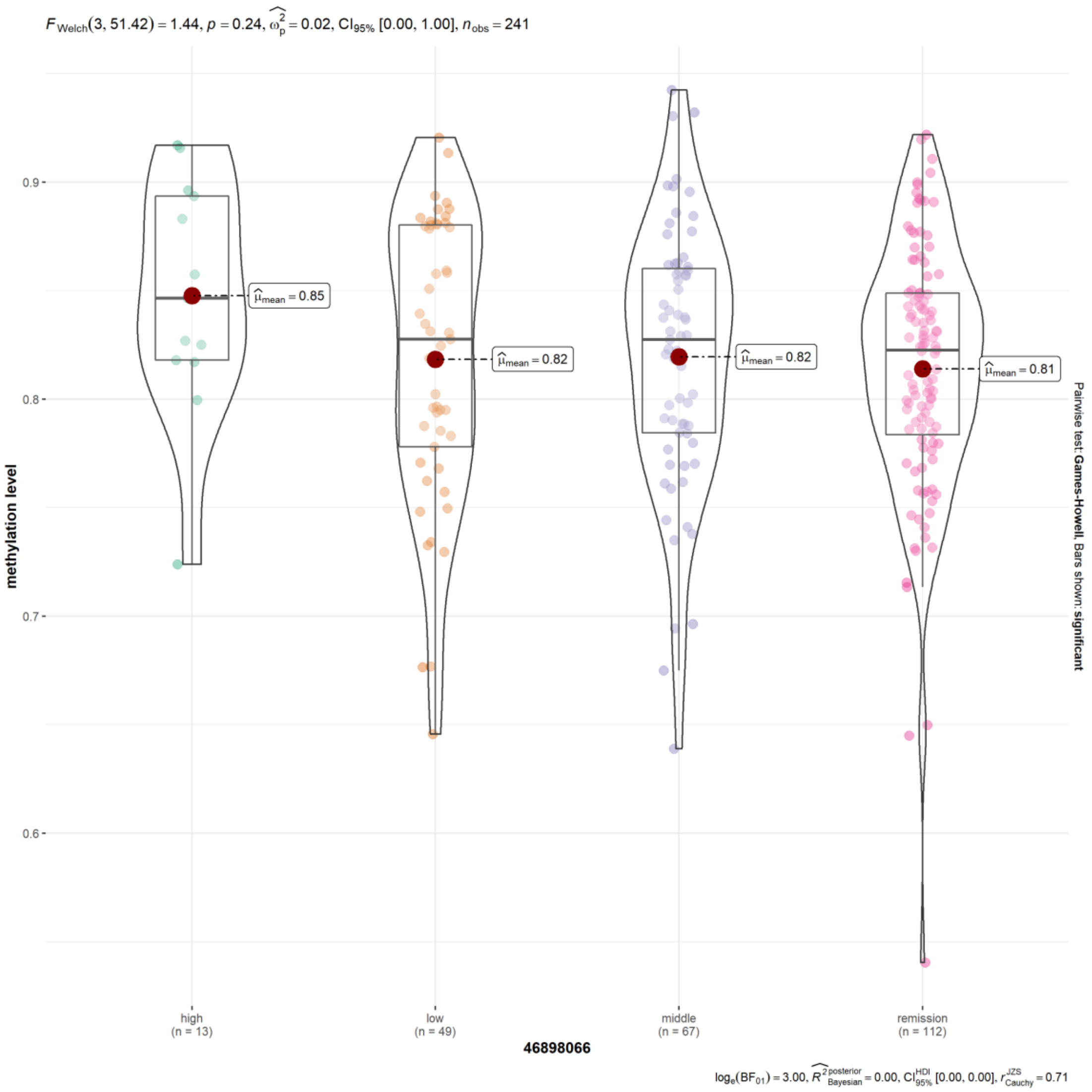

$F_{Welch}(3, 51.42) = 1.44$, $p = 0.24$, $\widehat{\omega_p^2} = 0.02$, $CI_{95\%}$ [0.00, 1.00], $n_{obs} = 241$
methylation level
0.9
0.8
0.7
0.6
$\hat{\mu}_{mean} = 0.85$
$\hat{\mu}_{mean} = 0.82$
$\hat{\mu}_{mean} = 0.82$
$\hat{\mu}_{mean} = 0.81$
high
(n = 13)
low
(n = 49)
middle
(n = 67)
remission
(n = 112)
46898066
Pairwise test: Games-Howell, Bars shown: significant
$\log_e(BF_{01}) = 3.00$, $\widehat{R^2}^{posterior}_{Bayesian} = 0.00$, $CI^{HDI}_{95\%}$ [0.00, 0.00], $r^{JZS}_{Cauchy} = 0.71$

## [DAS28-ESR]

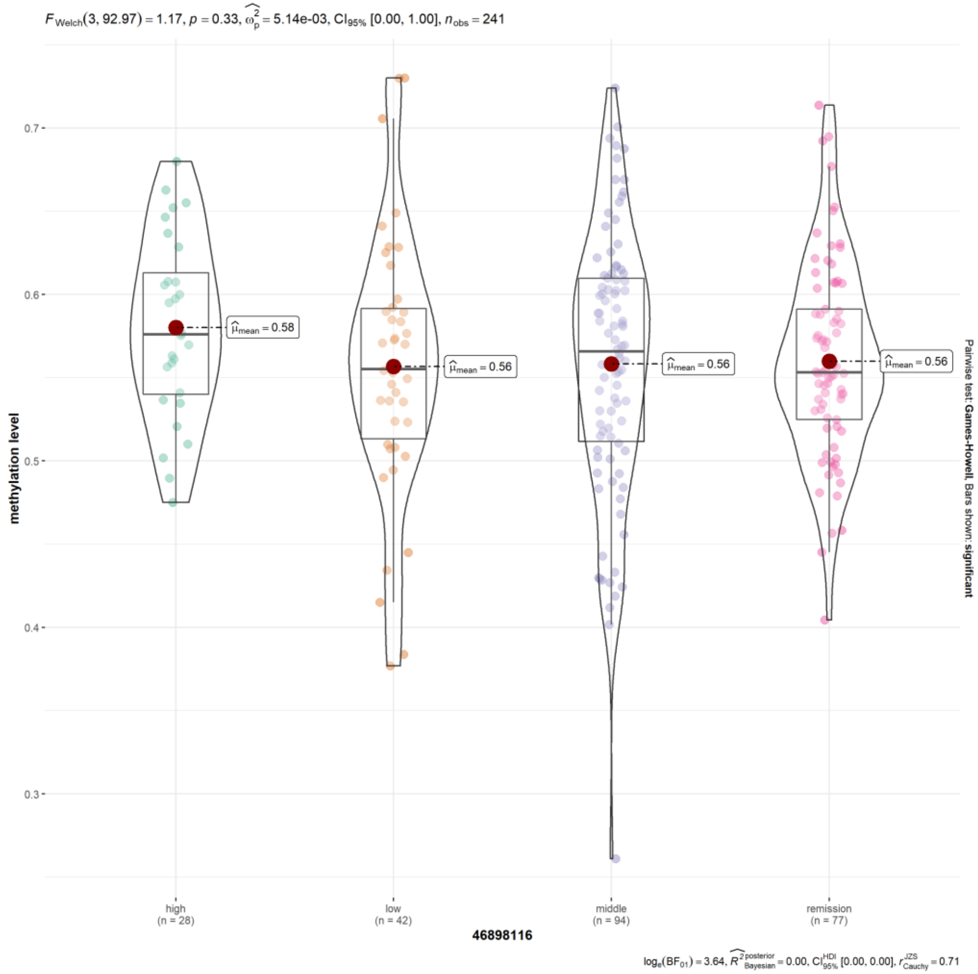

$F_{Welch}(3, 92.97) = 1.17, p = 0.33, \widehat{\omega_p^2} = 5.14e-03, CI_{95\%} [0.00, 1.00], n_{obs} = 241$
methylation level
0.3
0.4
0.5
0.6
0.7
$\hat{\mu}_{mean} = 0.58$
$\hat{\mu}_{mean} = 0.56$
$\hat{\mu}_{mean} = 0.56$
$\hat{\mu}_{mean} = 0.56$
high
(n = 28)
low
(n = 42)
middle
(n = 94)
remission
(n = 77)
46898116
Pairwise test: Games-Howell; Bars shown: significant
$\log_e(BF_{01}) = 3.64, \widehat{R^2}{}^{posterior}_{Bayesian} = 0.00, CI^{HDI}_{95\%} [0.00, 0.00], r^{JZS}_{Cauchy} = 0.71$

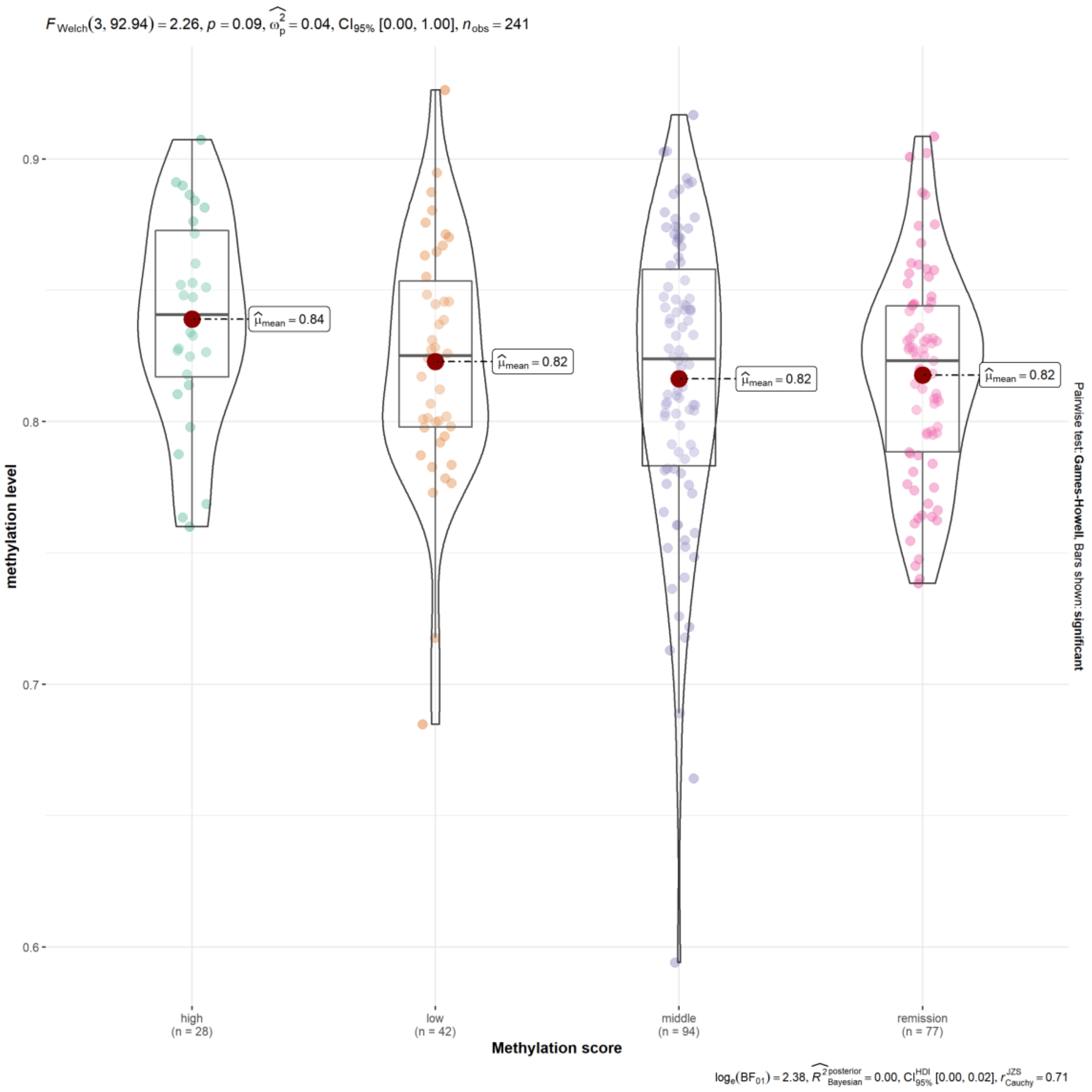

$F_{Welch}(3, 92.94) = 2.26$, $p = 0.09$, $\widehat{\omega_p^2} = 0.04$, $CI_{95\%}$ [0.00, 1.00], $n_{obs} = 241$
methylation level
0.9
0.8
0.7
0.6
$\widehat{\mu}_{mean} = 0.84$
$\widehat{\mu}_{mean} = 0.82$
$\widehat{\mu}_{mean} = 0.82$
$\widehat{\mu}_{mean} = 0.82$
high
(n = 28)
low
(n = 42)
middle
(n = 94)
remission
(n = 77)
Methylation score
Pairwise test: Games-Howell, Bars shown: significant
$\log_e(BF_{01}) = 2.38$, $\widehat{R^2}_{Bayesian}^{posterior} = 0.00$, $CI_{95\%}^{HDI}$ [0.00, 0.02], $r_{Cauchy}^{JZS} = 0.71$

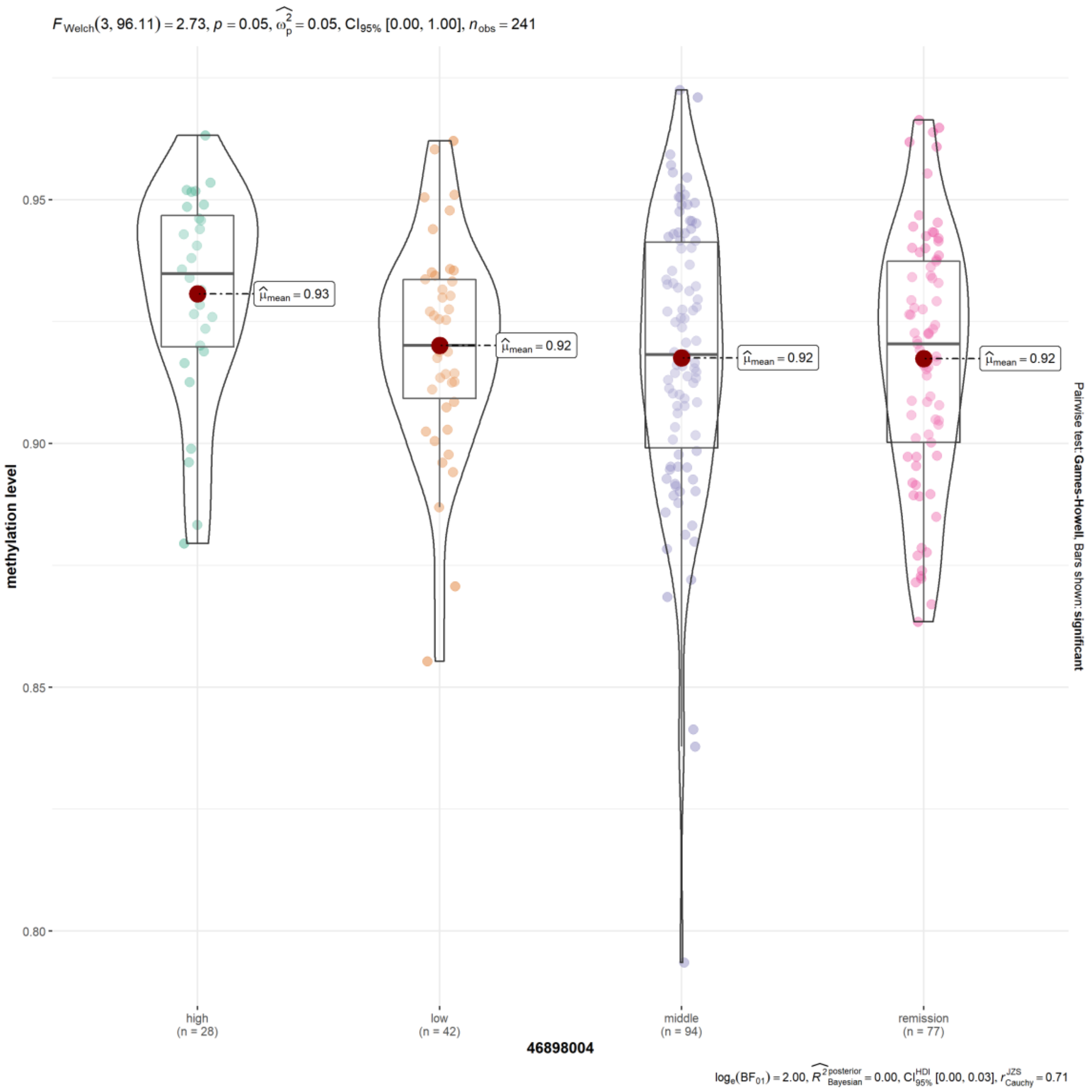

$F_{Welch}(3, 96.11) = 2.73$, $p = 0.05$, $\widehat{\omega_p^2} = 0.05$, $CI_{95\%}$ [0.00, 1.00], $n_{obs} = 241$
methylation level
0.95
0.90
0.85
0.80
$\hat{\mu}_{mean} = 0.93$
$\hat{\mu}_{mean} = 0.92$
$\hat{\mu}_{mean} = 0.92$
$\hat{\mu}_{mean} = 0.92$
high (n = 28)
low (n = 42)
middle (n = 94)
remission (n = 77)
46898004
Pairwise test: Games-Howell; Bars shown: significant
$\log_e(BF_{01}) = 2.00$, $\widehat{R^2}_{Bayesian}^{posterior} = 0.00$, $CI_{95\%}^{HDI}$ [0.00, 0.03], $r_{Cauchy}^{JZS} = 0.71$

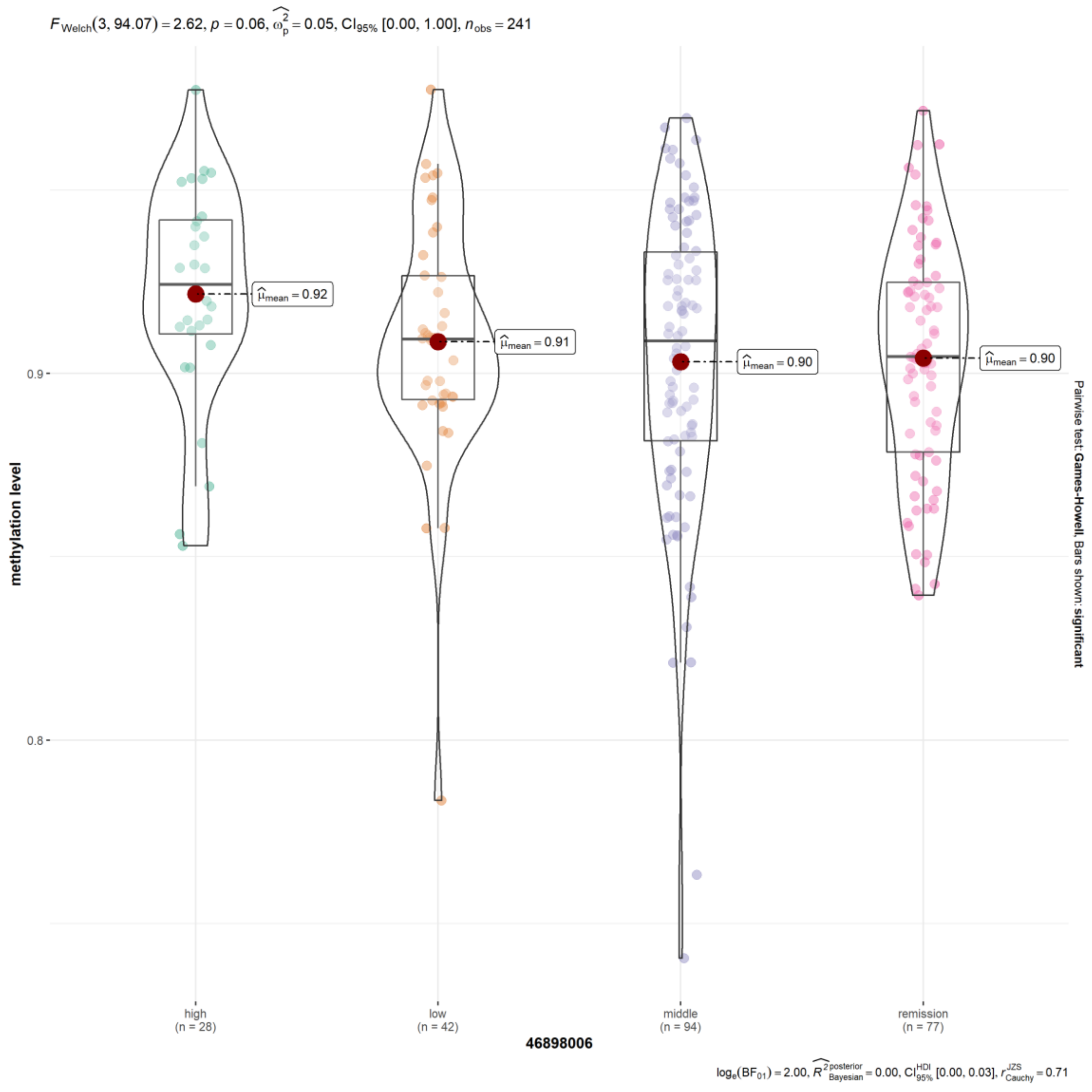

$F_{Welch}(3, 94.07) = 2.62$, $p = 0.06$, $\widehat{\omega_p^2} = 0.05$, $CI_{95\%}$ [0.00, 1.00], $n_{obs} = 241$
methylation level
0.9
0.8
$\hat{\mu}_{mean} = 0.92$
$\hat{\mu}_{mean} = 0.91$
$\hat{\mu}_{mean} = 0.90$
$\hat{\mu}_{mean} = 0.90$
high
(n = 28)
low
(n = 42)
middle
(n = 94)
remission
(n = 77)
46898006
Pairwise test: **Games-Howell**, Bars shown: **significant**
$\log_e(BF_{01}) = 2.00$, $\widehat{R^2}^{posterior}_{Bayesian} = 0.00$, $CI^{HDI}_{95\%}$ [0.00, 0.03], $r^{JZS}_{Cauchy} = 0.71$

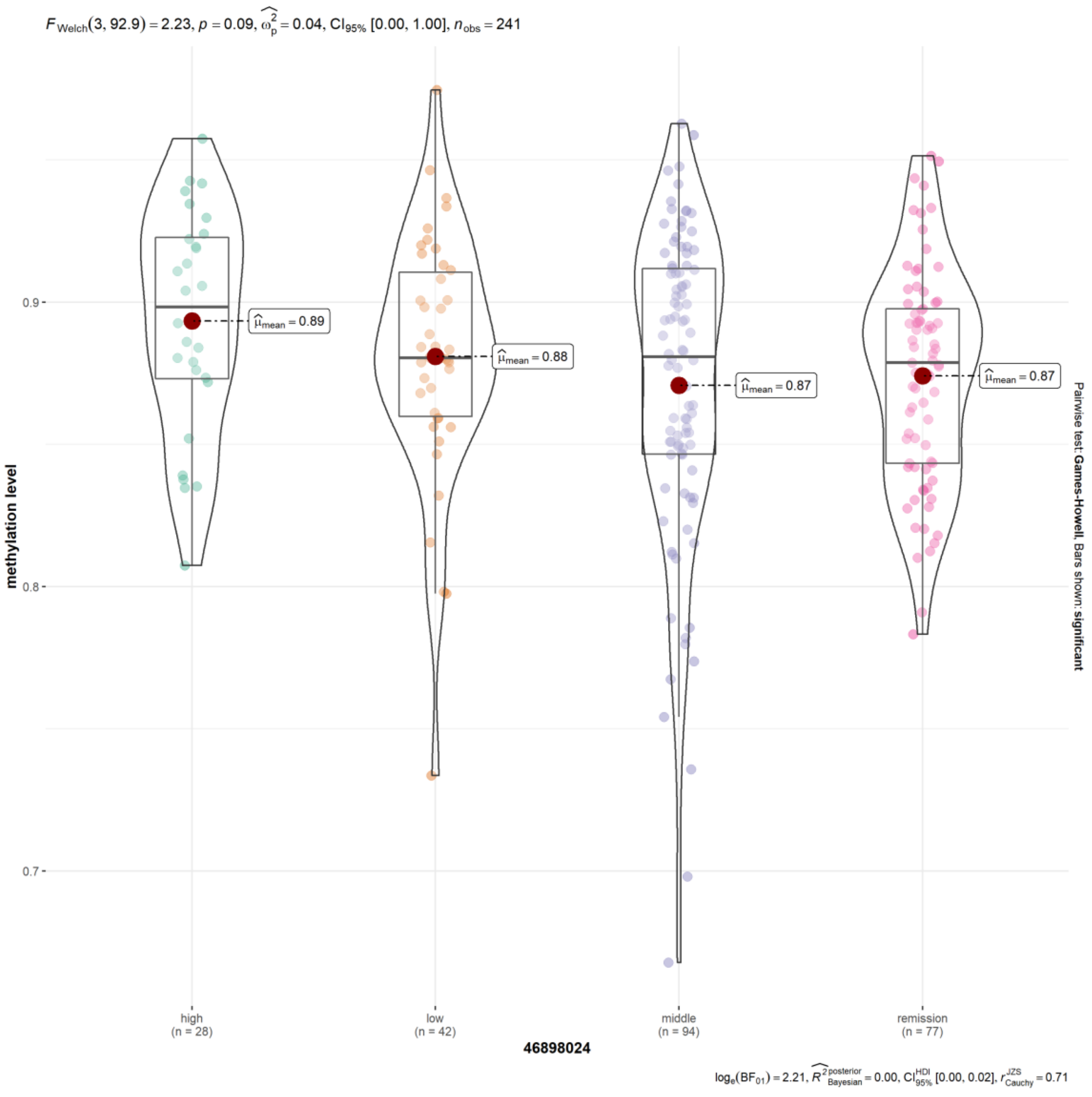

$F_{Welch}(3, 92.9) = 2.23$, $p = 0.09$, $\widehat{\omega_p^2} = 0.04$, $CI_{95\%}$ [0.00, 1.00], $n_{obs} = 241$
methylation level
0.9
0.8
0.7
$\hat{\mu}_{mean} = 0.89$
$\hat{\mu}_{mean} = 0.88$
$\hat{\mu}_{mean} = 0.87$
$\hat{\mu}_{mean} = 0.87$
high (n = 28)
low (n = 42)
middle (n = 94)
remission (n = 77)
46898024
Pairwise test: **Games-Howell**, Bars shown: **significant**
$\log_e(BF_{01}) = 2.21$, $\widehat{R^2}_{Bayesian}^{posterior} = 0.00$, $CI_{95\%}^{HDI}$ [0.00, 0.02], $r_{Cauchy}^{JZS} = 0.71$

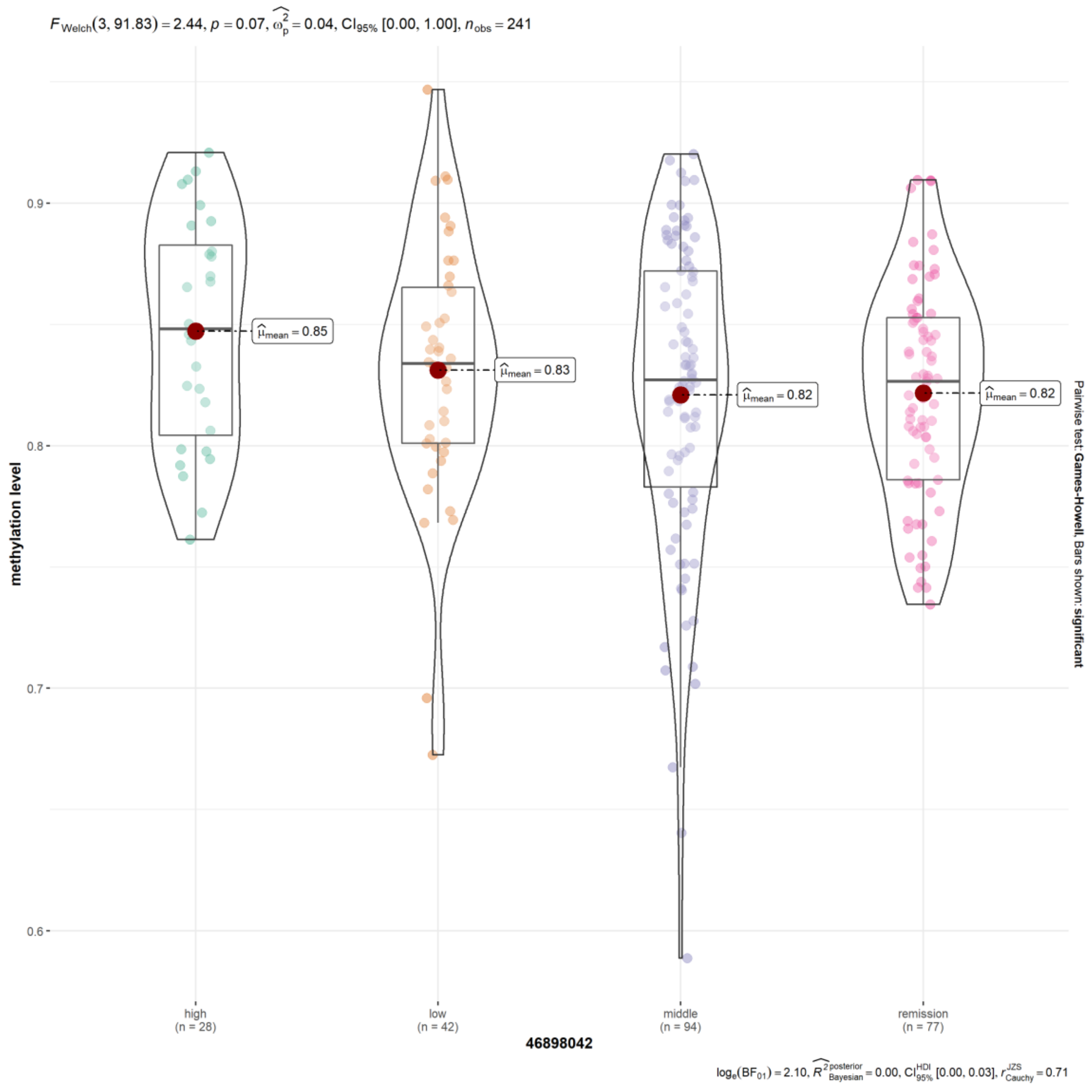

$F_{Welch}(3, 91.83) = 2.44$, $p = 0.07$, $\widehat{\omega_p^2} = 0.04$, $CI_{95\%}$ [0.00, 1.00], $n_{obs} = 241$
methylation level
0.9
0.8
0.7
0.6
$\hat{\mu}_{mean} = 0.85$
$\hat{\mu}_{mean} = 0.83$
$\hat{\mu}_{mean} = 0.82$
$\hat{\mu}_{mean} = 0.82$
high
(n = 28)
low
(n = 42)
middle
(n = 94)
remission
(n = 77)
46898042
Pairwise test: **Games-Howell**, Bars shown: **significant**
$\log_e(BF_{01}) = 2.10$, $\widehat{R^2}^{posterior}_{Bayesian} = 0.00$, $CI^{HDI}_{95\%}$ [0.00, 0.03], $r^{JZS}_{Cauchy} = 0.71$

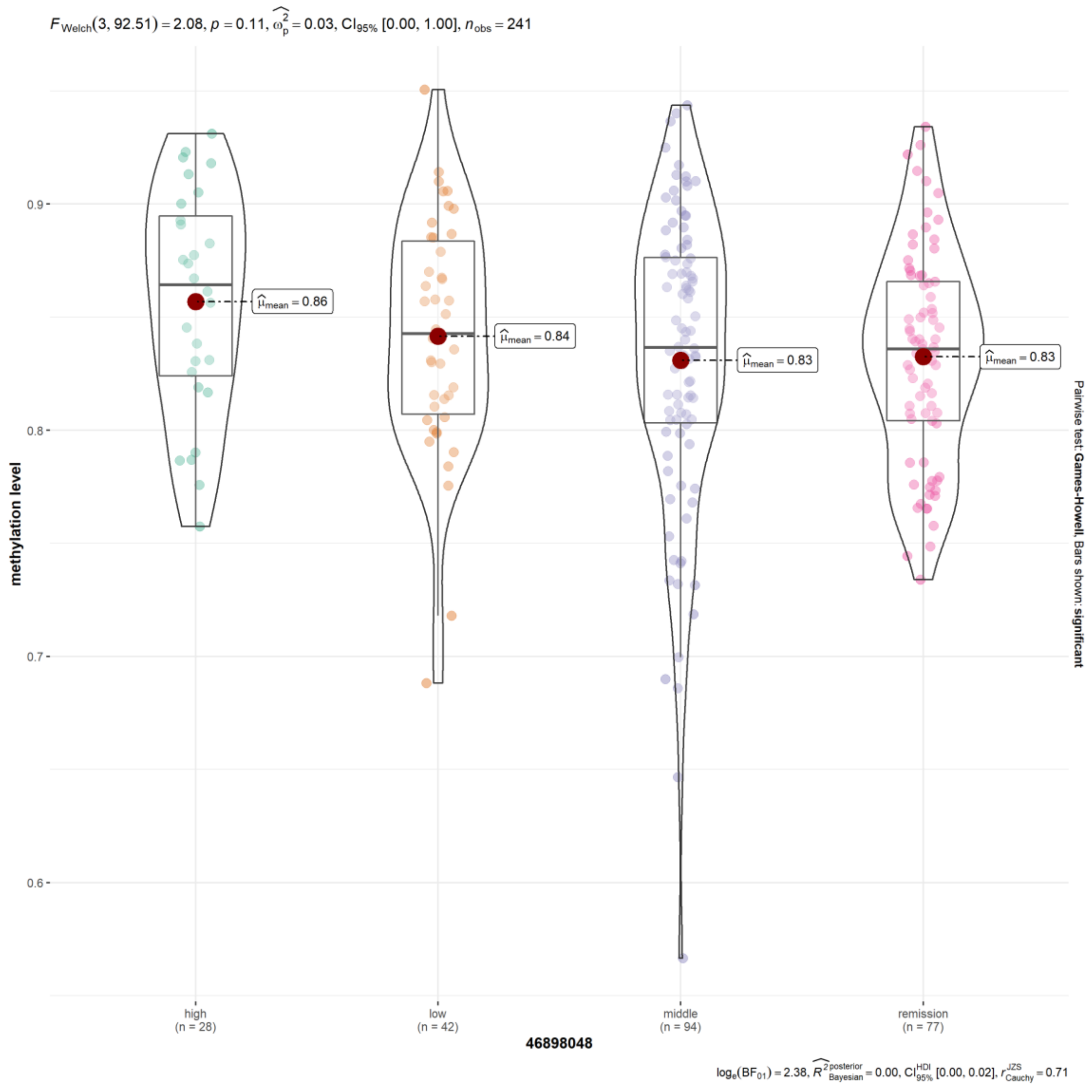

$F_{Welch}(3, 92.51) = 2.08$, $p = 0.11$, $\widehat{\omega_p^2} = 0.03$, $CI_{95\%}$ [0.00, 1.00], $n_{obs} = 241$
methylation level
0.9
0.8
0.7
0.6
$\hat{\mu}_{mean} = 0.86$
$\hat{\mu}_{mean} = 0.84$
$\hat{\mu}_{mean} = 0.83$
$\hat{\mu}_{mean} = 0.83$
high (n = 28)
low (n = 42)
middle (n = 94)
remission (n = 77)
46898048
Pairwise test: Games-Howell; Bars shown: significant
$\log_e(BF_{01}) = 2.38$, $\widehat{R^2}_{Bayesian}^{posterior} = 0.00$, $CI_{95\%}^{HDI}$ [0.00, 0.02], $r_{Cauchy}^{JZS} = 0.71$

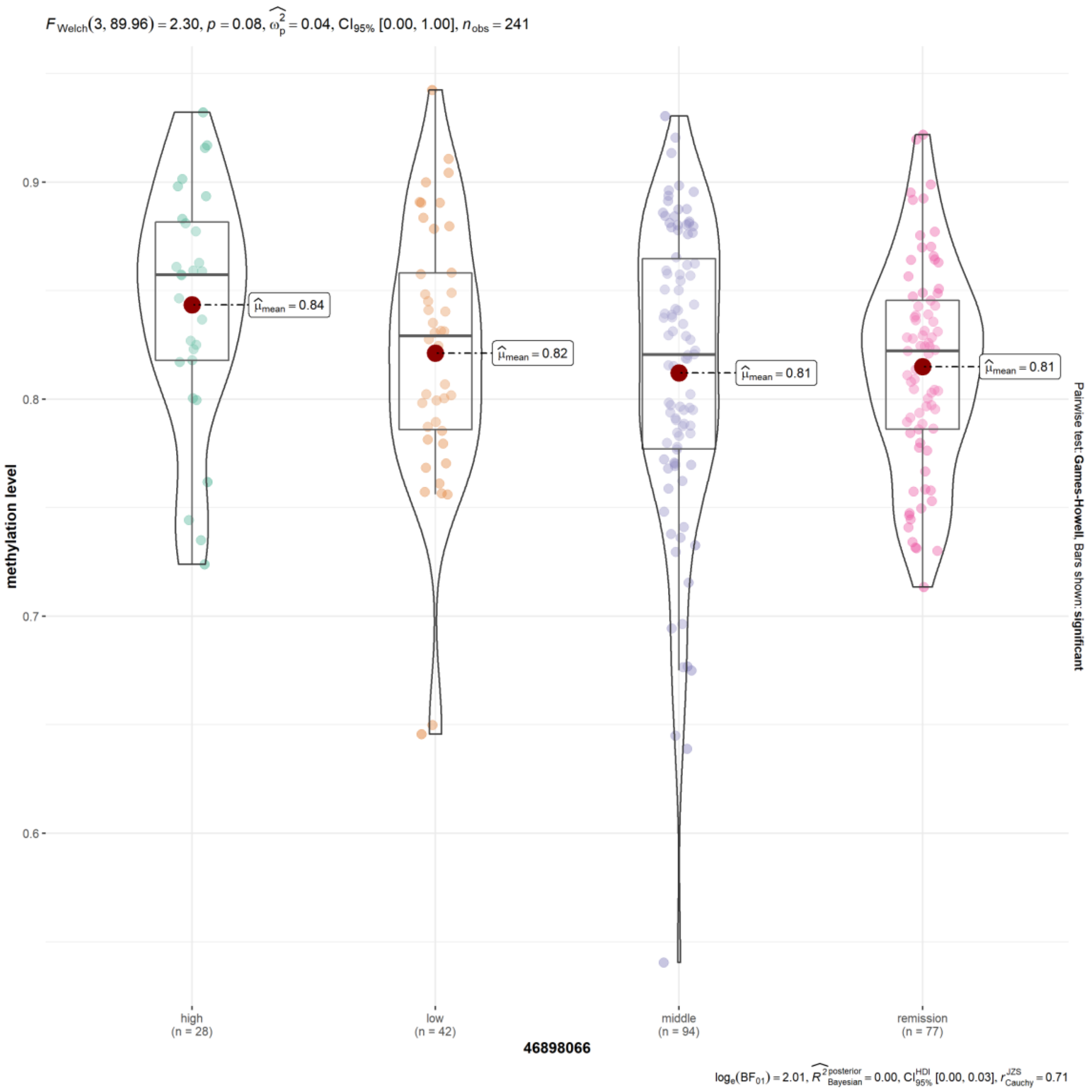

$F_{Welch}(3, 89.96) = 2.30$, $p = 0.08$, $\widehat{\omega_p^2} = 0.04$, $CI_{95\%}$ [0.00, 1.00], $n_{obs} = 241$
$\hat{\mu}_{mean} = 0.84$
$\hat{\mu}_{mean} = 0.82$
$\hat{\mu}_{mean} = 0.81$
$\hat{\mu}_{mean} = 0.81$
methylation level
0.9
0.8
0.7
0.6
high (n = 28)
low (n = 42)
middle (n = 94)
remission (n = 77)
46898066
Pairwise test: Games-Howell, Bars shown: significant
$\log_e(BF_{01}) = 2.01$, $\widehat{R^2}^{posterior}_{Bayesian} = 0.00$, $CI^{HDI}_{95\%}$ [0.00, 0.03], $r^{JZS}_{Cauchy} = 0.71$
